# Distinctive Genes Determine Different Intramuscular Fat and Muscle Fiber Ratios of the *longissimus dorsi* Muscles in Jinhua and Landrace Pigs

**DOI:** 10.1371/journal.pone.0053181

**Published:** 2013-01-03

**Authors:** Ting Wu, Zhenhai Zhang, Zhangqin Yuan, Li Jan Lo, Jun Chen, Yizhen Wang, Jinrong Peng

**Affiliations:** 1 Key Laboratory for Molecular Animal Nutrition, Ministry of Education, College of Animal Sciences, Zhejiang University, Hangzhou, China; 2 Department of Biochemistry and Molecular Biophysics, Columbia University, New York, New York, United States of America; 3 College of Life Sciences, Zhejiang University, Hangzhou, China; Institute of Zoology, Chinese Academy of Sciences, China

## Abstract

Meat quality is determined by properties such as carcass color, tenderness and drip loss. These properties are closely associated with meat composition, which includes the types of muscle fiber and content of intramuscular fat (IMF). Muscle fibers are the main contributors to meat mass, while IMF not only contributes to the sensory properties but also to the plethora of physical, chemical and technological properties of meat. However, little is known about the molecular mechanisms that determine meat composition in different pig breeds. In this report we show that Jinhua pigs, a Chinese breed, contains much higher levels of IMF than do Landrace pigs, a Danish breed. We analyzed global gene expression profiles in the *longissimus dorsi* muscles in Jinhua and Landrace breeds at the ages of 30, 90 and 150 days. Cross-comparison analysis revealed that genes that regulate fatty acid biosynthesis (e.g., fatty acid synthase and stearoyl-CoA desaturase) are expressed at higher levels in Jinhua pigs whereas those that regulate myogenesis (e.g., myogenic factor 6 and forkhead box O1) are expressed at higher levels in Landrace pigs. Among those genes which are highly expressed in Jinhua pigs at 90 days (d90), we identified a novel gene porcine *FLJ36031* (*pFLJ*), which functions as a positive regulator of fat deposition in cultured intramuscular adipocytes. In summary, our data showed that the up-regulation of fatty acid biosynthesis regulatory genes such as *pFLJ* and myogenesis inhibitory genes such as *myostatin* in the *longissimus dorsi* muscles of Jinhua pigs could explain why this local breed produces meat with high levels of IMF.

## Introduction

The Jinhua pig, named after Jinhua City in Zhejiag Province of eastern China, is a traditional, slow-growing breed with a high IMF content and is popular for its superior quality pork. Jinhua ham, a type of dry-cured ham produced from the meat of Jinhua pigs is the most famous brand name s in China and Jinhua ham was awarded first prize in the 1915 Panama International Merchandise Exhibition. Jinhua pigs show strong competency of oxidative metabolism and adipogenesis, which are believed to induce more satisfactory features in muscles, such as favorable meat color, marbling and flavor [1], [2]. In contrast, Landrace pigs, a commercial breed of Danish origin selected over many generations for rapid growth and enhanced carcass yield, show low activities of oxidative metabolism and adipogenesis which lead to trace amounts of fat depot. As a consequence, Landrace pigs produce comparatively less flavorful pork [3]–[5]. Thus, these two pig breeds serve as ideal models to study porcine growth performance and meat quality.

Skeletal muscle is the primary abundant porcine tissue that comprises 20to 50% of total body mass among different pig breeds, and is the main tissue responsible for meat production in pigs. It is also the major metabolic tissue and contributes up to 40% of the resting metabolic rate in adult pigs [6]. Skeletal muscle is a heterogeneous tissue that is composed of four muscle fiber types including oxidative (type I and IIa) and glycolic (type IIb) fibers [7]. Muscle with a higher content of oxidative fiber contains a higher percentage of lipids, capillaries, myoglobin and mitochondria [8]. Favorable meat traits such as color, flavor and tenderness have been found to be closely associated with a higher content of oxidative fibers in muscles [9], [10]. In addition, individuals with muscles that are abundant in oxidative fibers are less likely to produce pale, soft, exudative (PSE) meat. Therefore, understanding the molecular processes that govern the development and phenotypic characteristics of skeletal muscle is instrumental in the breeding of pigs with high meat quality.

Microarray technology can simultaneously examine the differential expression of a large number of genes in a given tissue [7], [11] and has been widely used to compare gene expression profiles for the identification of candidate genes responsible for relevant phenotypes [12]–[14]. For example, microarray analysis showed that sexual dimorphism of adipose tissue is determined by differentially regulated sex-specific genes regardless of diet [15]. In contrast, comparison of global gene expression profiles using Affymetrix Mu11K SubB containing 6516 probe sets revealed only 49 differentially expressed genes in the *quad* (white muscle) and the *soleus* (red muscle) [16]. Based on a home-made porcine cDNA microarray carrying 5,500 cDNA clones, Bai et al. identified 115 differentially expressed genes between the *psoas* (red muscle) and the *longissimus dorsi* (white muscle) of a 22-week-old Berkshire pig [17]. Over the past decade, a tremendous amount of porcine transcriptomics data has been obtained using the pig cDNA microarray [18]–[20], while the Affymetrix porcine genome array showed particularly superior performance for swine transcriptomics [21], [22]. However, reports on the comparison of global gene expression patterns in the skeletal muscles of different pig breeds at different developmental stages are lacking. In this study, a global gene expression profiling investigation was conducted to identify differentially expressed genes in *longissimus dorsi* muscles of Jinhua and Landrace pigs at three developmental stages using the Affymetrix GeneChip® Porcine Genome Array containing oligonucleotides representing approximately 23937 transcripts from 20201 porcine genes. We found that genes involved in adipogenesis and myogenesis were differentially expressed in Jinhua and Landrace pigs. To validate the potential utility of our microarray data, we characterized the expression and function of a novel gene, *pFLJ*, that is one of the genes up-regulated in Jinhua pigs at the age of d90 using both drug and gene-specific small interfering RNA (siRNA) treatment approaches in cultured intramuscular adipocyte precursor cells. Our results showed that knockdown of *pFLJ* expression down-regulated the genes involved in fat biosynthesis and reduced fat deposition, suggesting that pFLJ is a novel regulator of adipogenesis in the muscle.

## Results and Discussion

### Comparison of Carcass Traits and Meat Quality Features between Jinhua and Landrace Pig Breeds

**Figure 1:**
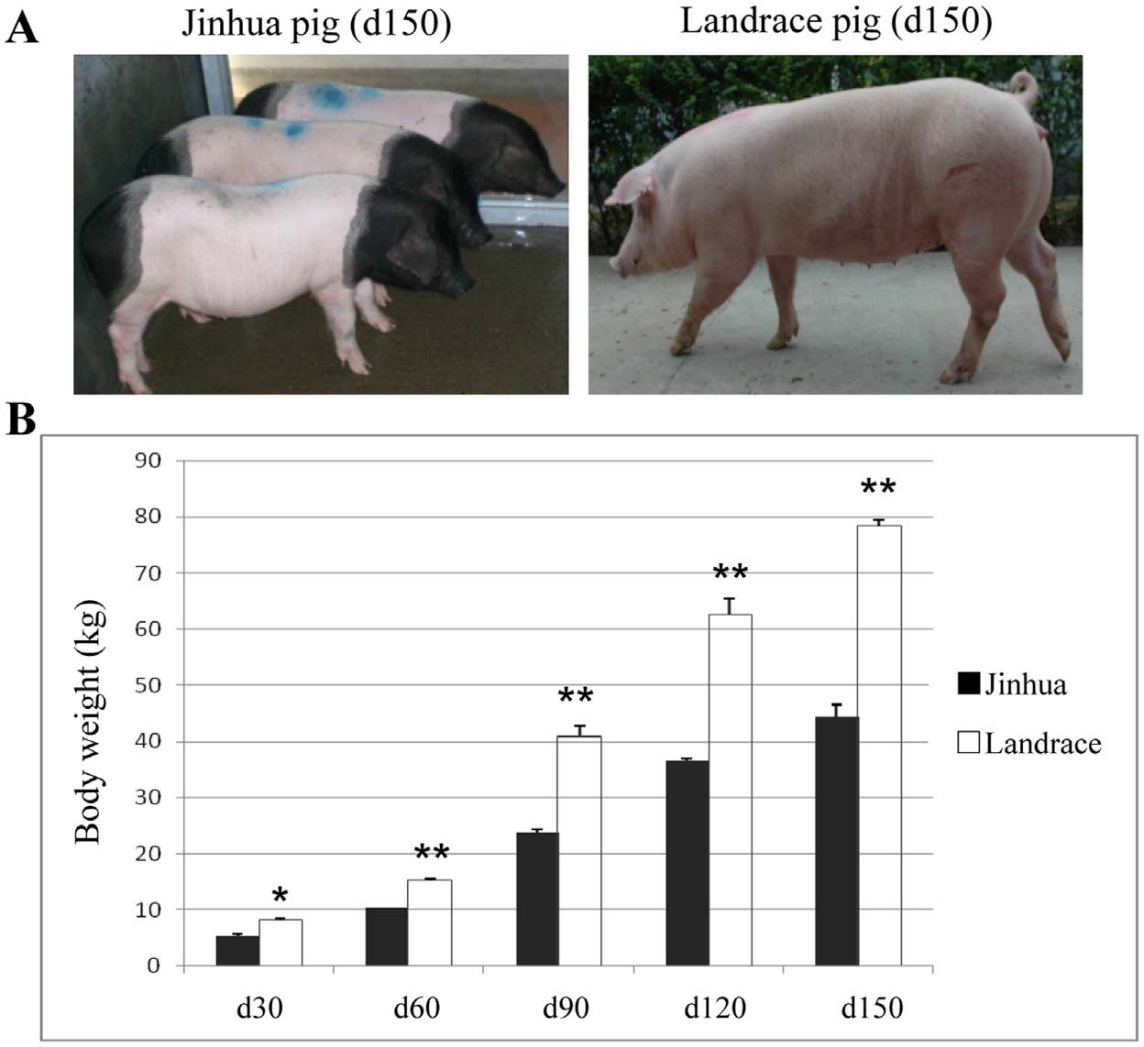
The Landrace breed grows faster than does the Jinhua breed. (A) Photographs showing three Jinhua pigs and one Landrace pig at d150. (B) Comparison of the body weight of Jinhua and Landrace pigs at the age of d30, d60, d90, d120 and d150, respectively. Landrace pigs gained weight much faster than Jinhua pigs. Pigs were slaughtered at around the age of d30, d90 and d150 (nine individuals per stage) and d60 and d120 (three individuals per stage) for each breed. Data are presented as means ± standard error. *P<0.05, **P<0.01.

The overall appearance of a typical adult Jinhua pig is very different from that of a Landrace pig (Figure 1A). Growth performance, meat quality and carcass traits in Jinhua and Landrace pigs at the same age (d30, d60, d90, d120, d150, days of age) were compared. Our results showed that from the age of d30 to d150, on average, Jinhua pigs gained approximately 40 kg in weight, while Landrace pigs gained about 70 kg (Figure 1B), demonstrating that the Jinhua were apparently growing more slowly than the Landrace. Analysis of the lean meat ratio (LMR) and loin meat area (LMA) showed that both were significantly lower in Jinhua pigs aged from d30 to d150 (Table 1). In contrast, Jinhua pigs exhibited significantly greater back fat thicknesses (BFT) and fat meat ratios (FMR) (Table 1, P<0.01). For example at d150, BFT and FMR in Jinhua pigs were about 2- and 2.4-fold higher, respectively (BFT: 23.7 mm in Jinhua versus 12.0 mm in Landrace; FMR: 32.4% in Jinhua versus 13.3% in Landrace) (Table 1).

**Table 1:** Determination of carcass traits and meat quality in Jinhua and Landrace pigs at the age stage of 30, 60, 90, 120 and 150 days age.^1^

Items		30		60		90		120		150	
		Jinhua(n = 9)	Landrace (n = 9)	Jinhua(n = 3)	Landrace (n = 3)	Jinhua(n = 9)	Landrace (n = 9)	Jinhua(n = 3)	Landrace (n = 3)	Jinhua(n = 9)	Landrace (n = 9)
BFT^2^ (mm)		9.33±1.23^A^	2.15±0.45^B^	11.00±1.07^A^	6.24±0.63^B^	20.03±0.91^A^	6.00±0.58^B^	21.90±0.76^A^	10.33±0.88^B^	23.70±0.92^A^	12.00±1.00^B^
FMR^3^ (%)		14.86±1.03^A^	6.70±0.94^B^	15.60±2.37^A^	7.01±0.24^B^	26.21±1.13^A^	7.67±0.54^B^	29.58±1.30^A^	8.36±0.29^B^	32.40±1.75^A^	13.26±1.26^B^
LMR^4^ (%)		44.27±0.52^A^	51.97±2.45^B^	47.19±1.28^A^	62.01±3.23^B^	42.73±1.13^A^	70.77±1.81^B^	40.78±0.64^A^	69.59±1.48^B^	41.01±1.48^A^	68.46±2.08^B^
LMA^5^ (cm^2^)		0.48±0.01	0.87±0.08	1.06±0.10	1.32±0.21	1.47±0.01^A^	3.19±0.05^B^	1.72±0.05^A^	4.04±0.69^B^	2.49±0.05^A^	5.40±0.21^B^
PH_45_ 6		5.99±0.19	6.12±0.12	6.26±0.25	6.50±0.11	6.31±0.06	6.52±0.09	6.15±0.02	6.46±0.13	6.39±0.04	6.32±0.36
Color^7^	l*	44.46±0.95^A^	41.11±0.63^B^	44.07±0.24	44.54±0.47	44.12±0.93	43.12±0.28	42.91±1.08	40.77±0.85	44.25±0.96^A^	39.77±0.26^B^
	a*	12.27±0.92	15.17±0.68	9.71±0.31	11.52±0.37	9.96±0.68	10.02±0.39	10.50±0.59	10.22±0.09	8.52±0.66^a^	10.33±0.39^b^
	b*	11.66±0.71	11.54±0.53	10.28±0.31	10.74±0.12	11.02±0.36	11.31±0.0.32	10.86±0.33	8.74±0.14	9.92±0.35	9.45±0.34

1 Results are presented as means ± standard error.

2 BFT = back fat thickness.

3 FMR = fat meat ratio.

4 LMR = lean meat ratio.

5 LMA = longissimus muscle area.

6 PH45 = pH value at 45 min postmortem.

7 Color = meat color. l*, a*, b* represent as lightness, redness and yellowness, respectively.

ab and AB Means with different superscripts of capital or lowercase letter at the same row of the same age are significantly different (P<0.05 or P<0.01).

It was previously reported that the Chinese Dahe pig breed displayed higher pH values (6.08) than the western crossbred Dawu sire line pig breed (5.79) 24 h postmortem [23]. A high pH value at 45 min *post mortem* (pH_45;_ 6.00–6.58) is known to correlate with a lower incidence of PSE meat [24]. We determined the pH_45_ values of both Jinhua and Landrace pigs at d30, d60, d90, d120 and d150, and found that they all ranged between 6.0- and 6.5 (Table 1), with no statistically significant differences between the two breeds. These results suggested that both breeds are less likely to produce PSE meat. Meat color parameters (L*, lightness; a*, redness; b*, yellowness) are used as an index of meat quality. Analysis of the color parameters showed that there was a significant tendency for the a* value in muscle *longissimus dorsi* to be lower in Jinhua pigs than in Landrace pigs at the age of d150 whilst L* and b* did not differ significantly between the two breeds (Table 1). However, several reports have shown that color parameters are not an adequate indicator of meat quality when the breed has a high IMF content [25]–[27].

### Jinhua Pigs have a High Content of IMF

**Figure 2:**
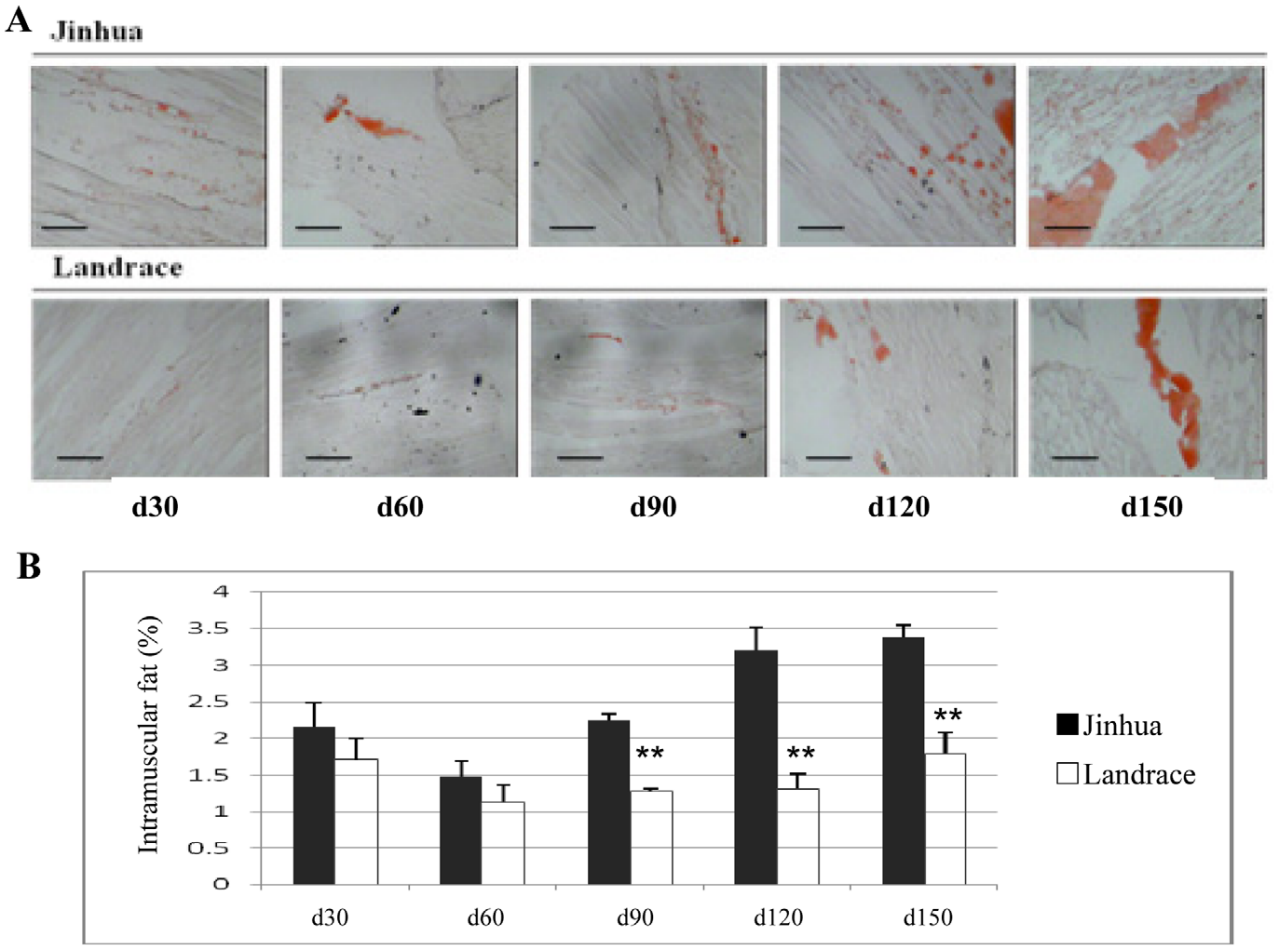
The Jinhua breed has a higher IMF content than the Landrace breed. (A) Oil Red O staining of *longissimus dorsi* muscles in Jinhua and Landrace pigs, respectively. Oil Red O stained IMF displayed a red color. (B) Comparison of IMF contents in *longissimus dorsi* muscles in Jinhua and Landrace pigs at the age of d30, d60, d90, d120 and d150, respectively. Pigs were slaughtered at around the age of d30, d90 and d150 (nine individuals per stage) and d60 and d120 (three individuals per stage) for each breed. Data are presented as means ± standard error. **P<0.01. Scale bars, 100 µm.

Oil red O staining showed that the distribution pattern of fat in the *longissimus dorsi* muscles in Jinhua pigs was more abundant than that in Landrace pigs (Figure 2A). Measurement of fat content revealed significant differences between Jinhua and Landrace pigs; Jinhua pigs showed a higher IMF content at all stages examined (Figure 2B). Notably, the IMF contents in Jinhua pigs showed a steady increase from d60 (1.48%), d90 (2.25%), d120 (3.20%) to d150 (3.38%) age stages, while that in Landrace pigs remianed relatively stable from d60 (1.13%), d90 (1.28%) to d120 (1.31%) with a only slight increase at d150 (1.79%) (Figure 2B, P<0.01). Interestingly, the IMF contents in both breeds decreased slightly from d30 to d60 (Figure 2B; P<0.01). Our results support previous findings that Jinhua pigs have greater BFT and IMF but lower LMR and LMA than Landrace pigs at the same age [28]. These characteristics define the superior flavor of Jinhua pork [29], [30].

### Global Gene Expression Profiles of *longissimus dorsi* Muscles in Jinhua and Landrace Pigs at d30, d90, and d150

By comparing features of growth rate (Figure 1B) and IMF content (Figure 2), we noted that the differences between Jinhua and Landrace pigs at d30, d90 and d150 three stages can be used to represent the early initiation, steady growth and maturation of myogenesis and adipogenesis in muscle, respectively. Based on this assumption, we decided to extract total RNAs from the *longissimus dorsi* muscles of both breeds at d30, d90, and d150 to perform microarray hybridization. Data obtained from 18 gene-chip hybridizations (nine gene-chips for each breed, three repeats for each stage) were processed according to the procedures described in Materials and Methods. We compared the global gene expression profiles of Jinhua pigs at d90 or d150 with that at d30. Our data showed that, in comparison with their expression at d30, a total of 419 differentially expressed genes were identified in *longissimus dorsi* muscles at d90, including 177 up-regulated genes (d90-up) and 242 down-regulated genes (d90-down) (Table 2; Table S1 and S2). A total of 490 differentially expressed genes were identified in *longissimus dorsi* muscles at d150, including 101 up-regulated (d150-up) genes and 389 down-regulated (d150-down) genes (Table 2; Table S3 and S4). Clustering analysis of microarray data [31] showed that, compared with their expression at d30, 37 genes were both d90-up and d150-up, 109 genes were d90-down and d150-down, two genes were d90-up but d150-down, and six genes were d30-down but d150-up (Table 2).

**Table 2:** Summary of the number of genes up- or down-regulated in *longissimus dorsi* muscles in Jinhua or Landrace pigs at age of d90 and d150.^1^

		No. of genes		No. of genes
Jinhua pigs	d90-up	177	d90-down	242
	d150-up	101	d150-down	389
	d90- & d150-up	37	d90- & d150-down	109
Landrace pigs	d90-up	106	d90-down	231
	d150-up	93	d150-down	387
	d90- & d150-up	11	d90- & d150-down	64
d90-up, Jinhuavs Landrace		2	d90-down, Jinhua vs Landrace	8
d150-up, Jinhuavs Landrace		6	d150-down, Jinhua vs Landrace	57

1 Number of genes was obtained by comparing the expression profiles between d30 and d90 or d30 and d150 in each breed. Details are listed in Tables S2 (d90-up in Jinhua pigs), S3 (d90-down in Jinhua pigs), S4 (d150-up in Jinhua pigs), S5 (d150-down in Jinhua pigs), S6 (d90-up in Landrace pigs), S6 (d90-down in Landrace pigs), S7 (d150-up in Landrace pigs), and S8 (d150-down in Landrace pigs).

In contrast, in *longissimus dorsi* muscles of Landrace pigs, 106 d90-up, 231 d90-down, 93 d150-up, 383 d150-down genes were identified, respectively, when compared with expression at d30 (Table 2; Table S5, S6, S7, S8). Clustering analysis of microarray data showed that, in comparison to expression at d30, 31 genes were both d90-up and d150-up, and 64 genes were d90-down and d150-down. Interestingly, no gene was found to be d90-up but d150-down or d90-down but d150-up (Table 2).

The fact that no or only a limited number of genes belonged to the d90-up/d150-down or d90-down/d150-up categories in both breeds suggests that the transcriptome operates sequentially to support the development of *longissimus dorsi* muscle during the d30 to d150 period. This provides a possible explanation for the continuous gain in muscle mass during this developmental window.

We also compared the d90-up and d90-down genes in Jinhua pigs with those of Landrace pigs. The results showed that only 0.7% of d90-up and 1.7% of d90-down genes were shared in these two breeds (Table 2). For d150-up and d150-down genes, only 3.2% of d150-up and 7.9% of d150-down genes were common to the two breeds (Table 2). These data clearly indicates that different genes are mobilized in these two breeds to govern the development of their respective *longissimus dorsi* muscles.

### Identification of Genes Differentially Expressed in Jinhua and Landrace Pigs during Muscle Development

The global expression profiles in *longissimus dorsi* muscles at d30, d90 and d150 in Jinhua pigs were compared with those in Landrace pigs at corresponding stages. A total of 375, 431 and 1195 genes were identified at d30, d90 and d150 age of stage, respectively, with at least 2.0-fold difference (P value<0.05) between two breeds (Table 3). Among these, 176, 276 and 525 genes corresponding to the stages of d30, d90 and d150 were up-regulated in Jinhua pigs (Jinhua-up genes) (Table 3; Table S9, S10, S11), and 199, 155 and 670 genes corresponding to the stages of d30, d90 and d150 were down-regulated (Jinhua-down genes) (Table 3; Table S12, S13, S14).

**Table 3:** Summary of the number of genes differentially expressed in *longissimus dorsi* muscles in Jinhuan and Landrace pigs at age of d30, d90 and d150.^1^

		No. of genes		No. of genes
30d	Jinhua-up	176	Jinhua-down	199
90d	Jinhua-up	276	Jinhua-down	155
150d	Jinhua-up	525	Jinhua-down	670

1 Number of genes was obtained by comparing the expression profiles between Jinhua and Landrace pigs of the same age. Details are listed in Tables S10 (d30 Jinhua-up), S11 (d90 Jinhua-up), S12 (d150 Jinhua-up), S13 (d30 Jinhua-down), S14 (d90 Jinhua-down), S15 (d150, Jinhua-down).

**Figure 3:**
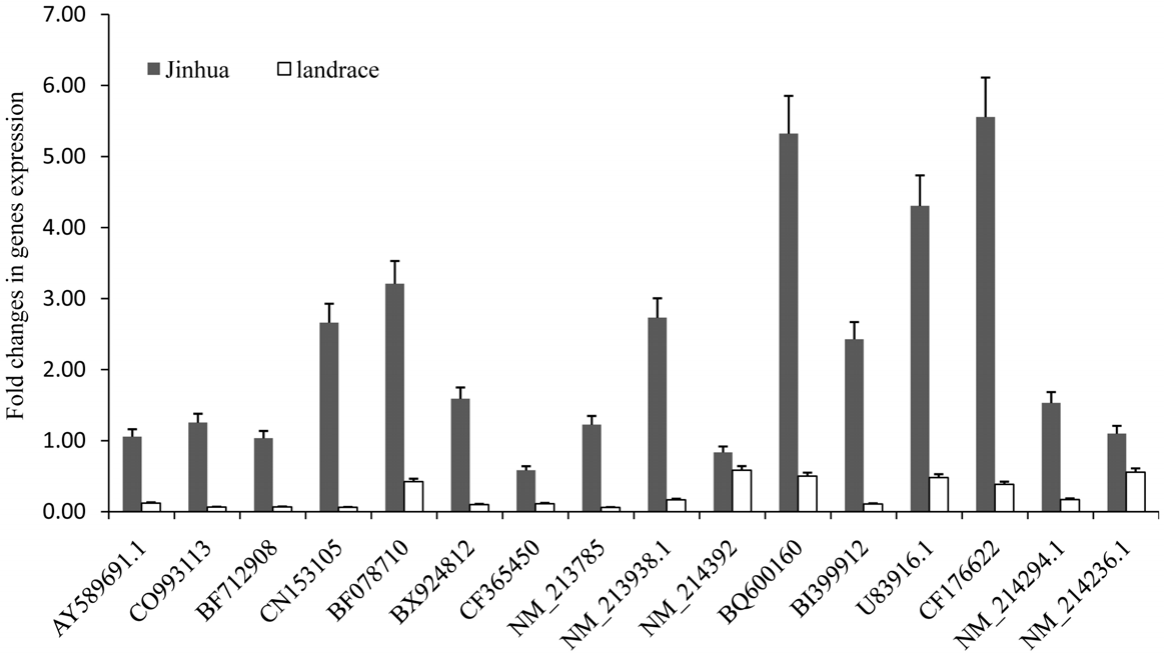
Validation of microarray data by qPCR. Validation by qPCR of 16 genes up-regulated in *longissimus dorsi* muscles of Jinhua pigs at d90 by qPCR. The qPCR values are shown as expression fold changes after normalization against the control 18s rRNA. Data are presented as means ± standard error. Gene ID was as shown. The full names of gene IDs representing AY589691.1, CO993113, BF712908, CN153105, BF078710, NM_213785.1, NM_213938.1, NM_214392.1, BI399912, U83916.1, NM_214294.1, NM_214236.1 are *adiponectin*, *heat shock 105kDa/110kDa protein 1*, *lipoprotein lipase*, *carbonic anhydrase II*, *leukemia inhibitory factor receptor*, *tissue factor*, *3-oxoacid CoA transferase 1*, *lysozyme, pyruvate dehydrogenase kinase*, *connective tissue growth factor, tropomodulin 3* and *myoglobin*. BX924812, CF365450, BQ600160, CF176622 are novel genes.

Among the differentially expressed genes identified by microarray in *longissimus dorsi* muscles of Jinhua and Landrace pigs at d90, 16 Jinhua-up genes (AY589691.1, CO993113, BF712908, CN153105, BF078710, BX924812, CF365450, NM_213785, NM_213938.1, NM_214392, BQ600160, BI399912, U83916.1, CF176622, NM_214294.1, NM_214236.1) were selected for validation by quantitative polymerase chain reaction (qPCR). Our results showed that with the exception of NM_214392 all of the selected genes were confirmed to be Jinhua-up genes (Figure 3). However, we notied that, although the patterns of differential expression of the examined genes were qualitatively similar between microarray and qPCR analysis (which shows the reliability of our microarray analysis), the fold changes obtained by the two approaches differed. We reasoned that this may be due to the greater accuracy of quantitation provided by qPCR compared with microarraysor to differences in the scope of magnitude of measurement of the two techniques [32].

### Adipose Deposition Related Genes are Differentially Activated in Jinhua and Landrace Pigs

A high IMF ratio is considered to be the major factor that contributes to the flavor of Jinhua meat. We noted that the IMF ratio in Jinhua pigs (2.25%) was ∼76% higher than that in Landrace pigs (1.28%) at d90 (Figure 2B), suggesting that, in addition to muscle development, IMF development program in Jinhua pigs must be activated at this time-point. We analyzed the differentially expressed genes in the two breeds at d30, d90 and d150 to elucidate the relationship between differential gene expression patterns and phenotypic differences in their *longissimus dorsi* muscles. Table 4, Table 5, and Table 6 (for pigs at d30, d90 and d150, respectively) listed the representative differentially expressed genes known to be related to adipose deposition and muscle development based on the OMIM database of National Center for Biotechnolgy Information (NCBI) (http://www.ncbi.nlm.nih.gov/omim/) and relevant publications that described their biological function.

**Table 4:** List of representative genes for adipogenesis and myogenesis differentially expressed in *longissimus dorsi* muscle in Jinhua and Landrace (L) pigs at d30.

Probe ID	Gene ID	Gene name	Gene Symbol	J/L Z score
**Adipose metabolism related genes_(Jinhua-up)**				
Ssc.16159.1.S1_at	NM_213781.1	stearoyl-CoA desaturase	SCD	5.97
Ssc.5538.1.S1_at	CN153105	similar to Carbonic anhydrase 2	LOC100154873	4.60
Ssc.1225.1.S1_at	CK455955	Similar to acetyl-Coenzyme A acyltransferase 1	LOC100152567	3.15
Ssc.17347.1.S1_at	NM_214349.1	pyruvate carboxylase	PC	2.78
Ssc.22959.1.S1_at	BX676168	phosphoenolpyruvate carboxykinase 1	CH242-37G9.2	6.63
Ssc.1147.2.S1_at	BF712908	Lipoprotein lipase	LPL	2.79
Ssc.6784.1.S1_at	AY686758.1	lipase, hormone-sensitive	LIPE	3.08
Ssc.18175.1.A1_at	CN166778	fatty acid synthase	FASN	6.13
Ssc.4360.1.A1_at	CB471223	fatty acid binding protein 3	FABP3	2.61
Ssc.18549.1.S1_at	AY589691.1	C1Q and collagen domain containing adiponectin	ADIPOQ	3.18
Ssc.11096.1.S1_at	NM_213938.1	3-oxoacid CoA transferase 1	OXCT1	2.59
Ssc.4021.1.S1_at	BG608754	1-acylglycerol-3-phosphate O-acyltransferase 1	SBAB-649D6.6	2.24
**Adipose metabolism related genes_(Jinhua-down)**				
Ssc.4292.1.S1_a_at	BF193243	similar to Peroxisomal biogenesis factor 19	LOC100154884	−2.94
Ssc.8779.1.A1_at	AJ658284	Similar to Apolipoprotein O-like	LOC100153260	−3.11
Ssc.10131.1.A1_at	BI399912	pyruvate dehydrogenase kinase, isozyme 4	PDK4	−3.65
Ssc.8139.1.S1_at	CB475937	Phytanoyl-CoA 2-hydroxylase	PHYH	−2.12
Ssc.21431.3.A1_s_at	CF789622	phosphoglycerate dehydrogenase	CH242-38B5.2	−2.95
Ssc.1942.1.S1_at	CN166665	lipin 1	LPIN1	−3.12
Ssc.9365.1.S1_at	NM_213883.1	insulin-like growth factor 2	IGF2	−2.65
Ssc.18585.1.S1_at	CN155220	beta glucuronidase	GUSB	−2.42
Ssc.13262.1.S1_at	BX924410	eukaryotic translation initiation factor 4E binding protein 1	EIF4EBP1	−2.90
Ssc.217.1.S1_at	NM_214060.1	esterase D	ESD	−2.39
**Muscle development related genes_(Jinhua- up)**				
Ssc.10256.1.A1_at	BI400362	phosphodiesterase 4B, cAMP-specific	PDE4B	3.72
Ssc.9096.1.S1_at	BF075680	MyoD family inhibitor domain containing	MDFIC	2.13
Ssc.9984.1.A1_at	BI399508	Kruppel-like factor 4	KLF4	2.09
Ssc.657.1.A1_at	NM_214214.1	chemokine (C-C motif) ligand 2	CCL2	3.78
Ssc.1901.1.A1_at	CO939491	cardiac muscle alpha actin 1	ACTC1	2.52
Ssc.9013.1.S1_at	NM_213878.1	calponin 1, basic, smooth muscle	CNN1	2.82
**Muscle development related genes_ (Jinhua- down)**				
Ssc.12901.1.A1_at	BI404128	similar to Peripheral plasma membrane protein CASK	LOC100153146	−2.32
Ssc.10232.1.A1_at	BI400288	similar to myosin regulatory light chain interacting protein	LOC100155795	−2.12
Ssc.21763.1.A1_at	CK456888	gamma Sarcoglycan	SGCG	−4.34
Ssc.715.1.S1_at	NM_214236.1	myoglobin	MB	−2.78
Ssc.16626.1.S1_at	AY188502.1	myogenic factor 6	MYF6	−2.03
Ssc.73.1.S1_at	NM_214014.1	forkhead box O1	FOXO1	−2.06
Ssc.7146.1.A1_at	BI182779	ATP-binding cassette, sub-family A, member 1	ABCA1	−3.57
Ssc.3715.3.A1_at	CA778869	Solute carrier family 7	SLC7A7	−2.19

**Table 5:** List of representative genes for adipogenesis and myogenesis differentially expressed in *longissimus dorsi* muscle in Jinhua and Landrace (L) pigs at d90.

Probe ID	Gene ID	Gene name	Gene Symbol	J/L Z score
**Adipose metabolism related genes_(Jinhua-up)**				
Ssc.1680.1.S1_at	CK451176	similar to WW domain containing E3 ubiquitin protein ligase 1	LOC100157283	2.04
Ssc.6238.2.S1_at	BI400300	similar to adenylate kinase 3	LOC100155691	2.59
Ssc.10131.1.A1_at	BI399912	pyruvate dehydrogenase kinase, isozyme 4	PDK4	2.16
Ssc.16335.1.S1_at	AY686760.1	lipoprotein lipase	LPL	3.05
Ssc.9637.1.S1_at	NM_213909.1	glutamate-ammonia ligase (glutamine synthetase)	GLUL	4.25
Ssc.31165.1.S1_at	BF191227	caveolin 2	CAV2	2.81
Ssc.1203.1.S1_at	AU055626	C1Q and collagen domain containing adiponectin	ADIPOQ	2.42
Ssc.142.1.S1_at	NM_214039.1	acyl-Coenzyme A dehydrogenase, C-4 to C-12 straight chain	ACADM	2.21
Ssc.11096.1.S1_at	NM_213938.1	3-oxoacid CoA transferase 1	OXCT1	2.31
Ssc.777.1.S1_at	AF414124.1	11-beta hydroxysteroid dehydrogenase isoform 1	HSD11B1	2.43
**Adipose metabolism related genes_(Jinhua-down)**				
Ssc.4292.1.S1_a_at	BF193243	similar to Peroxisomal biogenesis factor 19	LOC100154884	−2.22
Ssc.8779.1.A1_at	AJ658284	Similar to Apolipoprotein O-like	LOC100153260	−2.16
Ssc.6498.1.A1_at	BI360380	Mitogen-activated protein kinase 12	MAPK12	−2.00
Ssc.11557.1.A1_at	BI183574	ISG15 ubiquitin-like modifier	ISG15	−2.76
Ssc.15800.1.S1_at	NM_214099.1	insulin-like growth factor binding protein 5	IGFBP5	−2.09
Ssc.9365.2.S1_a_at	CK463136	insulin-like growth factor 2	IGF2	−2.42
**Muscle development related genes_(Jinhua- up)**				
Ssc.16664.1.A1_at	BG382637	Kruppel-like factor 9	KLF9	2.06
Ssc.9984.1.A1_at	BI399508	Kruppel-like factor 4	KLF4	2.96
Ssc.235.2.S1_at	M20160.1	calpastatin	CAST	2.22
Ssc.335.1.S2_at	AF188635.1	myostatin	MSTN	2.25
**Muscle development related genes_(Jinhua- down)**				
Ssc.715.1.S1_at	NM_214236.1	myoglobin	MB	−2.56
Ssc.11858.1.S1_at	CN163410	fibromodulin	FMOD	−2.41
Ssc.1901.1.A1_at	CO939491	cardiac muscle alpha actin 1	ACTC1	−4.48
Ssc.10297.1.S1_at	BX666372	capping protein (actin filament) muscle Z-line, beta	CAPZB	−2.23
Ssc.7538.1.S1_at	BQ604786	cadherin 1, type 1, E-cadherin (epithelial)	CDH1	−2.97

**Table 6:** List of representative genes for adipogenesis and myogenesis differentially expressed in *longissimus dorsi* muscle in Jinhua and Landrace (L) pigs at d150.

Probe ID	Gene ID	Gene Name	Gene Symbol	J/L Z score
**Adipose metabolism related_(Jinhua-up)**				
Ssc.2430.1.S1_at	CN156586	similar to solute carrier family 27	LOC100155567	2.18
Ssc.1294.3.S1_at	BX672817	similar to nitrilase 1	LOC100155270	2.07
Ssc.11149.2.S1_at	AW359358	Similar to carbonic anhydrase IX	LOC100152792	2.19
Ssc.3284.1.S1_at	NM_001001636.1	ribosomal protein L32	RPL32	2.65
Ssc.806.1.A1_at	AJ296004	ribosomal protein L23	RPL23	2.57
Ssc.939.1.S1_at	BP172489	ribosomal protein L12	RPL12	2.24
Ssc.13910.1.S1_at	BX667169	phenylethanolamine N-methyltransferase	PNMT	2.20
Ssc.37.1.S1_at	NM_214000.1	haptoglobin	HP	3.51
Ssc.18918.1.A1_at	CF365816	glutathione peroxidase 2	GPX2	4.59
Ssc.204.1.S1_at	NM_214423.1	cytochrome P450 3A29	CYP3A29	7.95
Ssc.825.1.S1_at	CK450245	claudin 7	CLDN7	3.10
Ssc.19471.1.A1_at	CF365558	Carboxylesterase 1 (monocyte/macrophage serine esterase 1)	CES1	2.25
Ssc.760.1.S1_at	NM_214246.1	carboxylesterase	CES3	2.92
Ssc.16162.1.S1_at	NM_214224.1	4-hydroxyphenylpyruvate dioxygenase	HPD	2.12
**Adipose metabolism related_(Jinhua-down)**				
Ssc.1008.1.A1_at	BF703815	wingless-type MMTV integration site family, member 10B	WNT10B	−2.11
Ssc.1049.1.S1_at	NM_213781.1	stearoyl-CoA desaturase	SCD	−7.06
Ssc.11488.2.S1_at	BF193243	similar to Peroxisomal biogenesis factor 19	LOC100154884	−2.43
Ssc.15928.1.A1_at	CF175359	insulin-like growth factor binding protein 7	IGFBP7	−2.36
Ssc.15950.1.S1_at	CN163405	insulin-like growth factor binding protein 6	IGFBP6	−3.32
Ssc.16169.1.S1_x_at	BP152514	insulin-like growth factor 2	IGF2	−3.74
Ssc.16473.1.S1_at	NM_214281.1	fumarate hydratase	FH	−2.40
Ssc.16671.1.S1_at	CB285696	fatty acid binding protein 2, intestinal	FABP2	−2.02
Ssc.17914.1.S1_at	CK461797	Cellular retinoic acid binding protein 1	LOC100169745	−3.29
Ssc.17991.1.A1_at	NM_214438.1	caveolin 1	CAV1	−2.00
Ssc.18061.1.A1_at	CF178743	calsarcin 1	LOC733663	−2.14
Ssc.18223.1.A1_at	BQ599486	C1q and tumor necrosis factor related protein 3	C1QTNF3	−3.46
Ssc.18296.2.S1_a_at	BF080387	ATP citrate lyase	ACL	−2.34
Ssc.18318.1.S1_at	BI401144	arachidonate 5-lipoxygenase-activating protein	ALOX5AP	−3.42
**Muscle development related_(Jinhua- up)**				
Ssc.13859.1.A1_at	CN069994	Unc-45 homolog B	UNC45B	2.47
Ssc.2464.1.S1_at	BI400766	Stanniocalcin 1	STC1	2.53
Ssc.18494.2.A1_at	CF180682	similar to ankyrin repeat domain 2 (stretch responsive muscle)	LOC100155185	3.74
Ssc.20874.3.A1_at	BP165311	similar to Alpha-centractin (Centrosome-associated actin homolog) (ARP1)	LOC100156619	2.23
Ssc.9781.1.S1_at	NM_213910.1	serpin peptidase inhibitor, clade E (nexin, plasminogen activator inhibitor type 1), member 1	SERPINE1	2.19
Ssc.16060.1.S1_at	AF128841.1	sarcolumenin precursor	CBPG	3.24
Ssc.21716.1.A1_at	BG834768	protein phosphatase 1 catalytic subunit alpha isoform	LOC733611	2.29
Ssc.23978.1.S1_at	BF080704	phosphatase and actin regulator 3	CH242-60A21.1	2.50
Ssc.27600.1.S1_at	AY579430.1	paired box 3	PAX3	2.62
Ssc.10199.3.S1_at	CF364321	dystrobrevin binding protein 1	DTNBP1	2.23
**Muscle development related_(Jinhua- down)**				
Ssc.12333.1.A1_at	CF366197	similar to fibronectin type III domain containing 1	LOC100154276	-3.22
Ssc.15316.1.S1_at	NM_001002824.1	myogenic differentiation 1	MYOD1	-2.41
Ssc.16494.1.A1_at	CB468993	fibronectin	FN1	-4.14
Ssc.16525.1.S1_at	CN163410	fibromodulin	FMOD	-5.33
Ssc.16584.1.A1_at	BI402879	fibrinogen-like 2	FGL2	-2.40
Ssc.1664.2.S1_at	NM_001001771.1	fibrillin 1	FBN1	-3.59

We first examined the genes related to adipose deposition. At d30, genes related to adipose deposition were clearly more active in Jinhua than in Landrace pigs (Jinhua-up genes) (Table 4). These include *stearoyl-CoA desaturase* (NM_213781.1), *acetyl-Coenzyme A acyltransferase 1* (CK455955), *lipoprotein lipase* (BF712908) [33]–[35], *hormone-sensitive lipase* (AY686758.1) [36]–[38], *fatty acid synthase* (CN166778) [39]–[41], *fatty acid binding protein 3* (CB471223) [42]–[44], *C1Q and collagen domain containing adiponectin* (AY589691.1) [45] and *1-acylglycerol-3-phosphate O-acyltransferase 1* (BG608754) etc. At d90 and d150, more adipose deposition-related genes were classified as Jinhua-up genes, including *caveolin 2* (BF191227) [46]–[48], *C-4 to C-12 straight chain acyl-Coenzyme A dehydrogenase* (NM_214039.1) [49], [50], *lipoprotein lipase* (AY686760.1) and *3-oxoacid CoA transferase 1* (NM_213938.1) [51] etc at d90 (Table 5), and *solute carrier family 27 member 4* (fatty acid transporter) (CN156586), *nitrilase 1* (BX672817) [52], *ribosomal protein L32* (NM_001001636.1), *ribosomal protein L23* (AJ296004) [53], *ribosomal protein L12* (BP172489), *claudin 7* (CK450245) and *carboxylesterase* (NM_214246.1) [54]–[56] etc at d150 (Table 6). These expression signatures correlate well with the fact that Jinhua pigs have a high IMF content.

In contrast, the *longissimus dorsi* muscles of Landrace pigs were found to express genes (Jinhua-down) such as *insulin-like growth factor 2* (NM_213883.1) [57], [58], *insulin-like growth factor binding protein 5* (NM_214099.1) [59], [60], *insulin-like growth factor binding protein 6* (CN163405) [59], *insulin-like growth factor binding protein 7* (CF175359) [61], *lipin 1* (CN166665) [62], [63] and *peroxisomal biogenesis factor 19* (BF193243) from d30 to d150 (Table 4, Table 5, Table 6). These genes are known to be involved in regulating fatty acid oxidation [64]–[66], suggesting that the *longissimus dorsi* muscles of Landrace pigs have stronger active in fatty acid oxidation than deposition.

### Muscle Development Related Genes are Differentially Expressed in Jinhua and Landrace Pigs

In contrast to the strong expression of genes related to adipose deposition, some key genes related to muscle development, including *myogenic factor 6* (AY188502.1), *forkhead box O1* (NM_214014.1) [67], [68], *γ-sarcoglycan* (CK456888) [69], [70], *myosin regulatory light chain interacting protein* (BI400288) and *peripheral plasma membrane protein CASK* (BI404128) [71], [72] were expressed at a lower level in Jinhua (Jinhua-down) than in Landrace pigs at d30 (Table 4). In addition, *myogenic differentiation 1* (NM_001002824.1) [73], [74] was also expressed at a lower level in Jinhua than in Landrace pigs at d150. In fact, Jinhua pigs appeared to express genes that slow down muscle development at d30 and d90. For example, *MyoD family inhibitor domain containing factor* (BF075680) [75] and *myostatin* (AF188635.1) [76], [77] were expressed at a higher level in Jinhua than inLandrace pigs at d30 and d90, respectively (Table 4 and Table 5). Consequently, many genes encoding muscle components were expressed at a lower level in Jinhua pigs (Jinhua-down) throughout the developmental stages of d30-d150, including *myoglobin* (NM_214236.1) [78], [79], *fibromodulin* (CN163410) [80], *β-capping protein* (actin filament) *muscle Z-line* (BX666372) [81], [82], *cardiac muscle alpha actin 1* (CO939491), *fibronectin type III domain containing 1* (CF366197) and *fibrinogen-like 2* (BI402879) (Table 4, Table 5, Table 6). This observation provides an explanation for the slow growth rate of Jinhua pigs.

Interestingly, some other factors which might be related to adipose deposition or muscle development were also found to be Jinhua-up, such as *Kruppel-like factor 4* (BI399508) [83], [84], *smooth muscle calponin 1* (NM_213878.1) and *chemokine* (C-C motif) *ligand 2* (NM_214214.1) at d30 (Table 4), *Kruppel-like factor 4* (BI399508), *Kruppel-like factor 9* (BG382637) [85] and *calpastatin* (M20160.1) [57], [86] at d90 (Table 5), and *ankyrin repeat domain 2* (stretch responsive muscle) (CF179329) [87], *stanniocalcin 1* (BP141278) [88], [89] and *Unc-45 homolog B* (CN069994) [90] at d150 (Table 6). It would be of great interest in future studies to determine how these factors contribute to the differences between Jinhua and Landrace pigs in growth rate and meat composition of the *longissimus dorsi* muscles.

### Transcription Factors and Signaling Molecules are Differentially Expressed in the *longissimus dorsi* Muscles in Jinhua and Landrace Pigs

Further analysis of the differentially expressed genes led us to identify a number of known transcription factors and signaling molecules that have not previously been reported to function in the development of *longissimus dorsi* muscles. Among these, we found that (*bone morphogenetic protein 1* (*BMP-1*), *regulator of G-protein signaling* (*RGS2*) and *proenkephalin* (*PENK*) were up-regulated whereas *four and a half LIM domains 3* (*FHL3*), *F-box protein 32* (*FBXO32*) and a gene similar to *CCAAT/enhancer-binding delta protein* (*LOC100153946*) were down-regulated in Jinhua pigs at 30d (Table 7). Transcription regulators *SWI/SNF related, matrix associated, actin dependent regulator of chromatin member 5* (*SMARCA5*), a gene similar to *T-box 3 protein* (*LOC100152741*) and *growth arrest and DNA-damage-inducible alpha* (*GADD45A*) were up-regulated while *selenoprotein X 1* (*SEPX1*), *homeobox protein A10* (*HOXD10A*) and *DNA cytosine-5-methyltransferase 3 alpha* (*DNMT3A*) were down-regulated in Jinhua pigs at d90 (Table 8). Interestingly, we noted that *BMP2* and *BMP receptor type 1B* (*BMPR1B*) which mediate BMP signaling were up-regulated while *secreted frizzled-related protein 4* (*SFRP4*) and *dickkopf homolog 3* (*DKK3*) which mediate Wnt signaling were down-regulated at d150 (Table 9), suggesting that key developmental signaling pathways are differentially mobilized in Jinhua and Landrace pigs. It will be of our great interest in the future to study how these transcription factors and signaling molecules control/regulate the distinct developmental events in Jinhua and Landrace pigs.

**Table 7:** List of genes encoding transcription factors and signaling molecules differentially expressed in *longissimus dorsi* muscle in Jinhua and Landrace pigs at d30.

Probe ID	Gene ID	Gene Name	Gene Symbol	J/L Z score
**Regulatory factors (Jinhua- up)**				
Ssc.16679.1.S1_at	BF079341	Similar to Bone morphogenetic protein 1 (BMP-1)	LOC100156461	2.44
Ssc.3139.1.A1_at	CK456262	Regulator of G-protein signaling 2, 24kDa	RGS2	2.63
Ssc.11281.1.A1_at	BI181438	proenkephalin	PENK	2.19
Ssc.396.1.S1_a_at	NM_214119.1	diazepam binding inhibitor (GABA receptor modulator, acyl-Coenzyme A binding protein)	DBI	2.59
Ssc.9707.1.A1_at	BX666261	BTG family, member 2	BTG2	2.32
**Regulatory factors (Jinhua- down)**				
Ssc.27892.2.S1_at	BX916748	Zinc finger, AN1-type domain 5	ZFAND5	−2.06
Ssc.7980.2.A1_at	BQ599924	similar to Zinc finger protein 22	LOC100156567	−2.10
Ssc.29341.1.A1_at	CO954104	similar to F-box and leucine-rich repeat protein 4	LOC100156082	−2.65
Ssc.23226.1.S1_at	CK452343	similar to E2F-associated phosphoprotein	LOC100153549	−2.14
Ssc.10025.3.S1_at	BI118416	similar to CCAAT/enhancer-binding delta protein	LOC100153946	−2.38
Ssc.22958.1.S1_a_at	CK457158	Similar to BTB (POZ) domain containing 1	LOC100154013	−2.55
Ssc.3931.1.S1_at	NM_213946.1	four and a half LIM domains 3	FHL3	−2.18
Ssc.4368.3.S1_at	BP463181	F-box protein 32	FBXO32	−4.87

**Table 8:** List of genes encoding transcription factors and signaling molecules differentially expressed in *longissimus dorsi* muscle in Jinhua and Landrace pigs at d90.

Probe ID	Gene ID	Gene Name	Gene Symbol	J/L Z score
**Regulatory factors (Jinhua- up)**				
Ssc.10918.1.A1_at	CK464481	TP53RK binding protein	TPRKB	2.02
Ssc.12878.1.S1_at	CB287966	SWI/SNF related, matrix associated, actin dependent regulator of chromatin, subfamily a, member 5	SMARCA5	2.68
Ssc.1913.1.A1_at	CN163609	slowmo homolog	CH242-247L10.6	2.40
Ssc.6578.1.S1_at	BI467852	similar to T-box 3 protein	LOC100152741	2.11
Ssc.26039.1.S1_at	BX926726	similar to RAR-related orphan receptor A	LOC100156637	2.00
Ssc.3654.1.A1_at	CK463456	membrane-associated ring finger (C3HC4) 6	6-Mar	2.45
Ssc.20913.1.S1_at	CN161066	growth arrest and DNA-damage-inducible, alpha	GADD45A	2.37
Ssc.4368.3.S1_at	BP463181	F-box protein 32	FBXO32	2.34
**Regulatory factors (Jinhua- down)**				
Ssc.15738.1.S1_at	CF176266	similar to Transmembrane emp24 domain-containing protein 3 precursor (Membrane protein p24B)	LOC100152423	−2.30
Ssc.1303.1.S1_at	CK455870	similar to Leukocyte elastase inhibitor (LEI) (Serpin B1) (LNPI)	LOC100155145	−2.25
Ssc.3004.2.S1_at	BI182015	similar to Chromosome 9 open reading frame 16	LOC100152322	−2.21
Ssc.5520.1.S1_at	CK462523	Selenoprotein X, 1	SEPX1	−3.08
Ssc.101.1.S1_at	NM_214023.1	secreted phosphoprotein 1	SPP1	−2.66
Ssc.26254.1.S1_at	BX926970	Homeobox protein A10	HOXD10A	−2.73
Ssc.1704.1.S1_at	BX915676	alpha DNA (cytosine-5-) -methyltransferase 3	DNMT3A	−2.05

**Table 9:** List of genes encoding transcription factors and signaling molecules differentially expressed in *longissimus dorsi* muscle in Jinhua and Landrace pigs at d90.

Probe ID	Gene ID	Gene Name	Gene Symbol	J/L Z score
**Transcriptors_(Jinhua- up)**				
Ssc.810.1.S1_at	AY550058.1	scavenger receptor class B member 2	Scarb2	2.52
Ssc.11352.1.A1_at	BI185713	karyopherin alpha 7 (importin alpha 8)	KPNA7	2.35
Ssc.15865.1.A1_at	AY010069.2	karyopherin alpha 3 (importin alpha 4)	KPNA3	2.49
Ssc.20913.1.S1_at	CN161066	growth arrest and DNA-damage-inducible, alpha	GADD45A	3.07
Ssc.66.1.S3_at	CO950299	bone morphogenetic protein receptor, type IB	BMPR1B	2.10
Ssc.4190.1.S1_at	CA779719	bone morphogenetic protein 2	BMP2	2.19
**Transcriptors_(Jinhua- down)**				
Ssc.10160.1.A1_at	BG609515	transcription factor AP-2 gamma (activating enhancer binding protein 2 gamma)	CH242-255C19.1	-2.44
Ssc.1020.1.S1_at	AJ583828.1	toll-like receptor 1	TLR1	−2.04
Ssc.10245.2.A1_a_at	CN163698	tissue factor pathway inhibitor	LOC100155068	−2.47
Ssc.10822.1.S1_at	CK455045	Similar to tumor suppressor candidate 3	LOC100156093	−2.68
Ssc.11131.1.S1_at	CF795993	similar to Sushi repeat-containing protein SRPX	LOC100156108	−3.67
Ssc.11310.2.A1_at	BQ600663	similar to pleckstrin 2	LOC100154251	−2.19
Ssc.11559.2.A1_at	CN032097	similar to PDZ and LIM domain 2	LOC100152859	−2.14
Ssc.11618.2.S1_at	BE235724	similar to neuritin	LOC100154738	−6.44
Ssc.11862.1.A1_at	BP150958	similar to KLC4 protein	LOC100157157	−2.37
Ssc.12963.1.S2_at	CK457158	Similar to BTB (POZ) domain containing 1	LOC100154013	−2.50
Ssc.13079.2.S1_at	CB286263	similar to Baiap2l2 protein	LOC100154063	−2.15
Ssc.140.1.S1_at	BG382598	secreted frizzled-related protein 4	SFRP4	−3.55
Ssc.17141.1.A1_at	CO949346	dickkopf homolog 3	DKK3	−2.15
Ssc.18231.2.S1_at	BI399410	AXL receptor tyrosine kinase	AXL	−2.78
Ssc.1850.1.A1_at	CO938780	angiopoietin-like 2	ANGPTL2	−3.22

### *pFLJ* Encodes a Novel Protein and is Highly Expressed in the *longissimus dorsi* Muscle of Jinhua Pigs at d90

**Figure 4:**
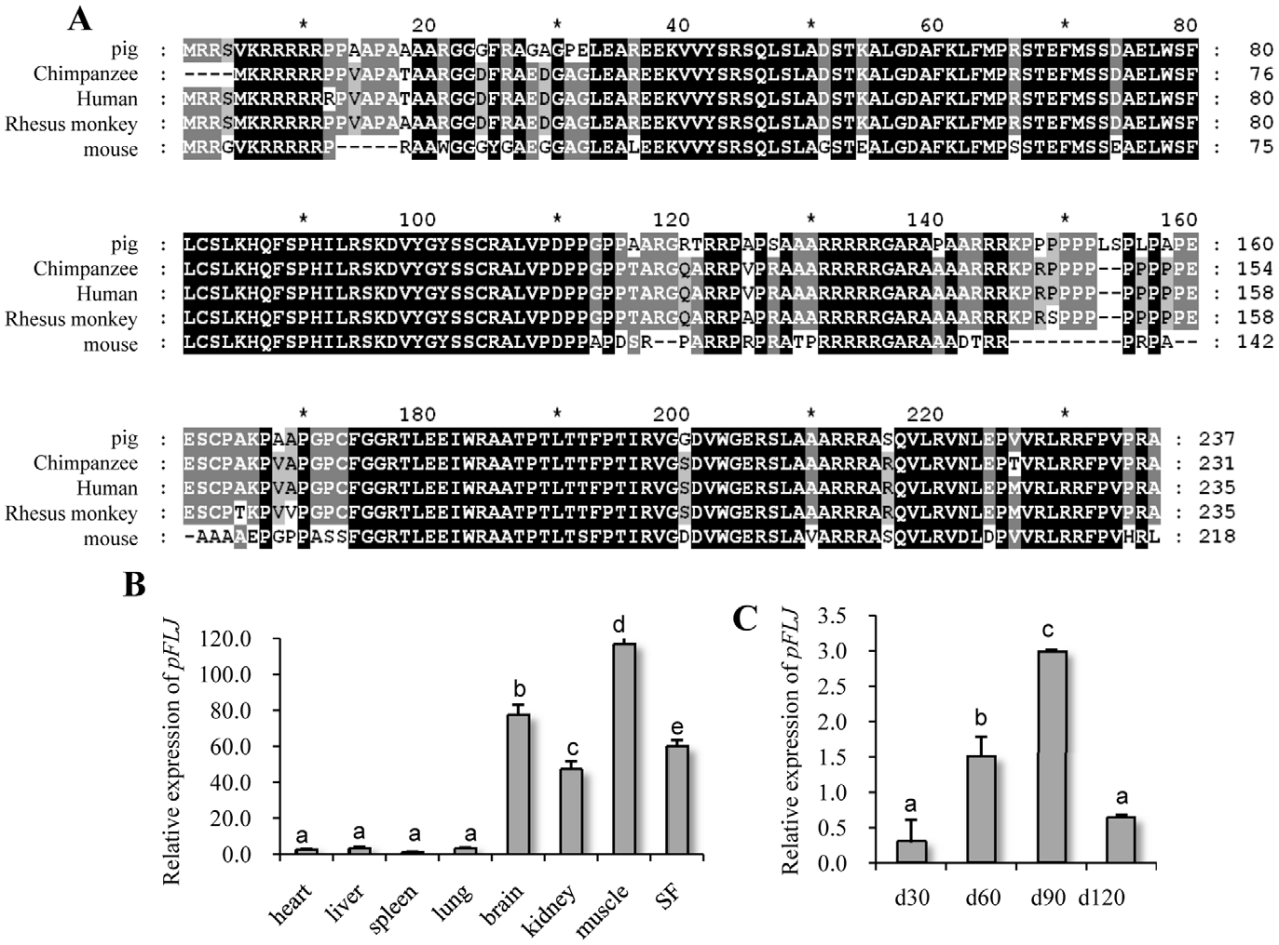
*pFLJ* encodes a novel protein and is highly expressed in *longissimus dorsi* muscle in Jinhua pigs at d90. (A) Alignment of amino acid sequences of FLJ homologues from pig (XP_003130310), chimpanzee (XP_001162764), human, rhesus monkey and mouse using the CLUSTAL X programme. (B and C) qPCR analysis of *pFLJ* expression in different organs/tissues (B) or in *longissimus dorsi* muscles in Jinhua at different stages as shown (C). The qPCR values are shown as expression fold changes after normalization against the control 18s rRNA. Data are presented as means ± standard error. Gene ID was as shown. ^ab^ means every two columns with different letters are significantly different (P<0.05).

The microarray data allowed us to search for novel genes involved in the adipogenesis process in muscles. We noted that one unknown gene corresponding to an expressed sequence tag (EST) with accession number BI184304 was expressed at a much higher level in Jinhua than in Landrace at d90. We cloned the full length cDNA corresponding to BI184304 through 5′- and 3′-rapid amplification of cDNA ends (RACE; data not shown) and found that this gene encodes a previously uncharacterized protein named FLJ in humans [91]. A database search revealed that FLJ is highly conserved among different species and pig FLJ (pFLJ) shares 93%, 83%, 92% and 92% homology with human, mouse, chimpanzee and rhesus monkey FLJ, respectively (Figure 4A).

qPCR was performed to examine the expression of *pFLJ* in different organs/tissues in Jinhua pigs. Our results showed that *pFLJ* is expressed at high levels in the brain, kidney, *longissimus dorsi* muscle and subcutaneous fatty tissue (SF) but at a much lower level in the heart, liver, spleen and lung, demonstrating that *pFLJ* is differentially expressed in pigs (Figure 4B). We then examined the expression of *pFLJ* in the *longissimus dorsi* muscles in Jinhua pigs at d30, d60, d90 and d120. Our results showed that the transcript levels of *pFLJ* sharply increased from d30 to d90, peaked at d90 and then decreased to a lower level at d120 (Figure 4C), thus *pFLJ* exhibits a dynamic expression pattern during skeletal muscle development.

### pFLJ is a Positive Regulator of Fat Deposition in Intramuscular Adipocytes

**Figure 5:**
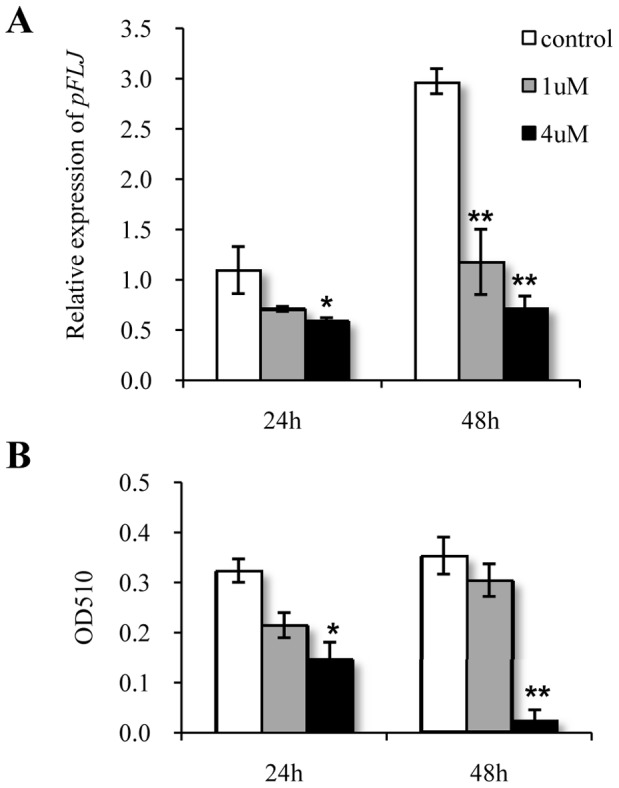
SR141716 down-regulates *pFLJ* expression and inhibits fat deposition in cultured intramuscular adipocytes. (A and B) qPCR analysis of pFLJ expression (A) and measurement of total triglyceride (B) in cultured adipocytes 24 and 48 hours after SR141716 treatment. The qPCR values are shown as expression fold changes after normalization against the control 18s rRNA. Data are presented as means ± standard error. Gene ID was as shown. Cells were stained with Oil-Red O to determine lipid accumulation (total triglyceride). *: P<0.05, **: P<0.01.

Because its expression levels and its dynamic expression pattern in the *longissimus dorsi* muscle differ between Jinhua and Landrace pigs, we wondered whether pFLJ might be involved in the process of adipogenesis. To address this question, we first established a protocol to culture intramuscular adipocyte precursor cells *in vitro*. These cells could be successfully induced to differentiate into adipocytes at 4 days, as judged easily by Oil Red staining (data not shown). qPCR revealed that *pFLJ* was expressed at a higher level in the differentiated adipocytes (data not shown). SR141716 (rimonabant, an antagonist of cannabinoid receptor 1 of mammals and commonly used as an inhibitor for fat deposition) was added to the cultured intramuscular adipocytes and the expression of *pFLJ* and fat contents were determined at 24- and 48-hour after treatment, respectively. Our data showed that SR14716 significantly down-regulated the transcript levels of *pFLJ* (Figure 5A) and fat deposition (Figure 5B) 48 hours after treatment.

**Figure 6:**
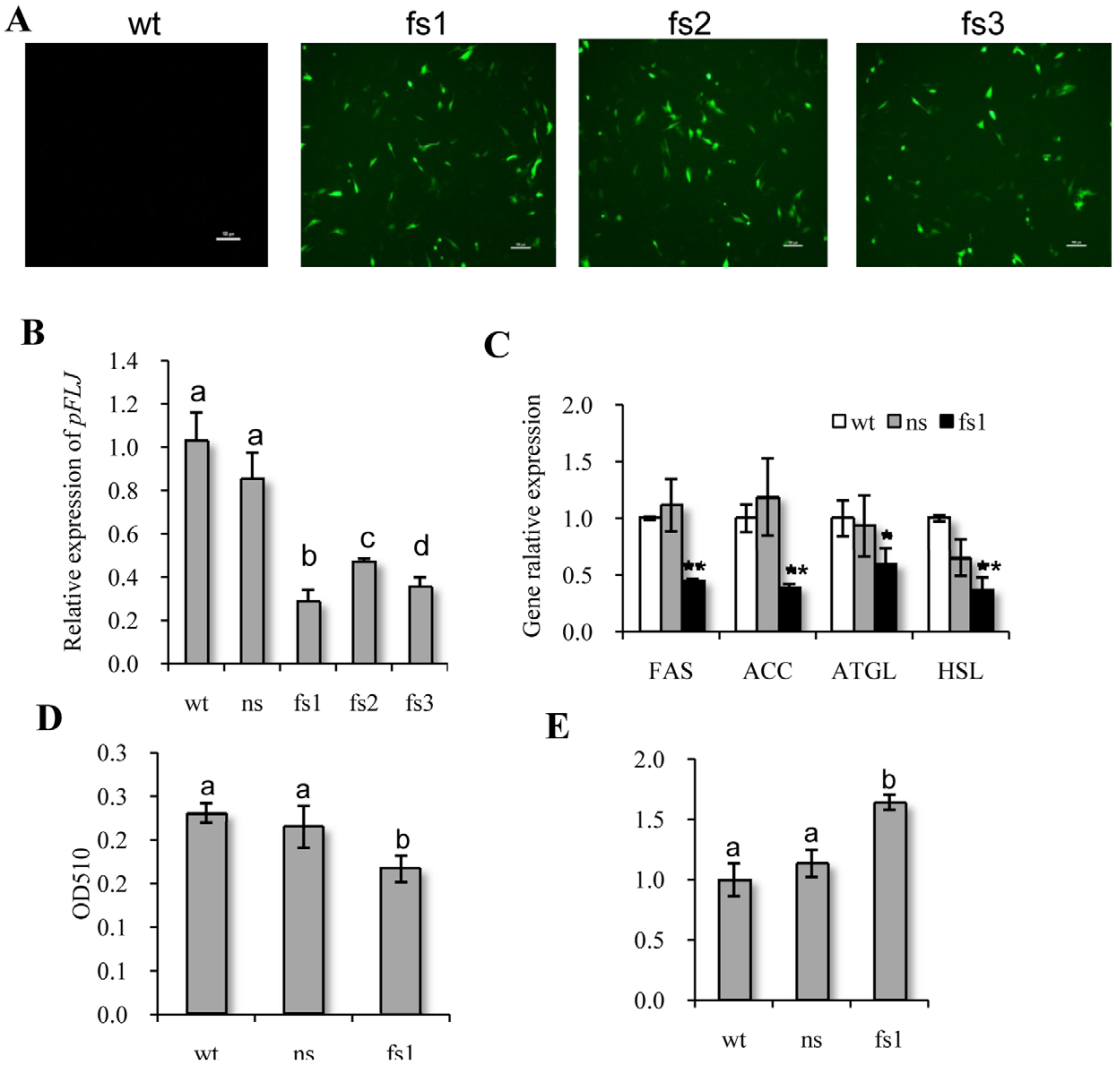
pFLJ functions as a positive regulator of fat deposition in intramuscular adipocytes. (A) Cell images to verify transfection efficiency. Cells were transfected with pSilencer TM 4.1-CMV neo plasmids carrying the sequences fs1, fs2 and fs3. Transfection efficiency was assessed by expression of the reporter gene *EGFP* (green color) harbored by the plasmid. (B) qPCR analysis of *pFLJ* expression in cultured adipocytes 24 hours after siRNA treatment. fs1, fs2 and fs3: pFLJ specific siRNAs; ns: negative control siRNA. (C) qPCR analysis of *FAS*, *ACC*, *ATGL* and *HSL* in cultured adipocytes treated with fs1 siRNA. The qPCR values are shown as expression fold changes after normalization against the control 18s rRNA. Data are presented as means ± standard error. *: P<0.05, **: P<0.01 (C and D) Measurement of total triglyceride (as before) in the cultured adipocytes or free glycerol (the free glycerol release was normalized to total cellular protein and expressed relative to the control group) in the culture medium 36 hours after treatment with fs1 siRNA. ^ab^ means every two columns with different letters are significantly different (P<0.05).

The above data suggest a probable role of pFLJ in fat deposition. To test this supposition, three siRNAs (fs1, fs2, fs3) were designed to targets the *pFLJ* transcript specifically. qPCR showed that these three siRNAs efficiently knocked down the transcript levels of *pFLJ* in cultured intramuscular adipocytes (Figure 6A), with fs1 showing the strongest effect at 36 hours after treatment (Figure 6B). These cultured cells were treated with *pFLJ* siRNA sf1 and control siRNA NS and the contents of total triglyceride (fat) in the treated cells and free glycerol in the culture medium 36 hours after treatment were measured. We found that the total triglyceride level was significantly down-regulated (Figure 6D), which in turn resulted in an elevation in free glycerol levels in the medium (Figure 6E). We then examined the transcript levels of *fatty acid synthase* (*FAS*), *acetyl-CoA carboxylase* (*ACC*), *adipose triglyceride lipase* (*ATGL*) and *hormone sensitive lipase* (*HSL*) in the siRNA treated cells. *FAS* and *ACC* encode two key enzymes for the synthesis of fat while *ATGL* and *HSL* gene products are responsible for the hydrolysis of fat. We found that transcript levels of all four genes were significantly down-regulated (Figure 6C). We therefore concluded that pFLJ is a positive regulator of fat deposition in cultured intramuscular adipocytes, probably by regulating the expression of genes that are essential for fat biosynthesis.

### Conclusion

In summary, our results revealed that genes that regulate adipogenesis and myogenesis are differentially expressed in Jinhua and Landrace pigs, with Jinhua pigs expressing higher levels of adipogenesis genes and Landrace expressing higher levels of myogenesis genes. More importantly, from the microarray data, a novel gene, *pFLJ*, was identified as a positive factor in the regulation of fat deposition in intramuscular adipocytes. *pFLJ* exhibited dynamic spatial and temporal expression patterns in Jinhua pigs, with high expression in the muscle at d90. Down-regulation of *pFLJ* by either drug treatment or siRNA-mediated gene knockdown reduced fat deposition concomitantly with the down-regulation of genes responsible for fat biosynthesis. This observation strongly suggests that up-regulation of *pFLJ* together with other factors (e.g *myostatin*, a myogenesis inhibitory gene) in the *longissimus dorsi* muscles of Jinhua pigs might play a key role in determining their high rate of IMF. Future efforts will be needed to determine the functional mechanism of pFLJ in this process. Therefore, transcriptomes for adipogenesis and myogenesis in the *longissimus dorsi* muscles are mobilized differentially in Jinhua and Landrace pig to produce meats with different ratios of muscle fiber to intracellular fat.

## Materials and Methods

### Ethics Statement

This study did not involve non-human primates. All experiments described in the study were performed in full accordance with the guidelines for animal experiments released by the National Institute of Animal Health with a permit (License No: GB/T 14925-94).

### Animals

Sixty six castrated Jinhua (Jinhua II breed) and Landrace (Danish breed) pigs were raised and had *ad libitum* access to commercial diets (nutrients levels according to the NRC) under similar conditions during the whole experimental period. Nine individual pigs from each breed at each stages (d30, d90 and d150) and three individuals per breed at each stages (d60 and d120) were slaughtered. The *longissimus dorsi* muscles at the last rib were collected after exsanguinations and were subsequently divided into four portions for use in the measurement of intramuscular fat, determination of meat color, determination of pH values, and isolation of total RNA. For RNA extraction, the excised samples were directly frozen in liquid nitrogen and stored at −80°C until use.

### Determination of Meat Quality

At each stage (d30, d60, d90, d120 and d150), experimental pigs were individually weighed and average bodyweights of all pigs of each breed at each stage were obtained. The BFT value was averaged from the fat thickness values measured on the first rib, last rib and the last lumbar vertebrae for each individual pig using a sliding caliper (Messschieber 0–150 mm mit Momentfeststellung Nonius 1/20 mm, Wollschlaeger). The FMR or LMR were calculated as the ratio of weight of fat meat or lean meat to the total weight of fat meat, lean meat, skin and bone of the left ham. LMA was determined by tracing its surface area at the 10th rib and calculating the area using a planimeter (Planix 5.6, Tamya Digital Planimeter, Tamaya Tecnics Inc., Tokyo, Japan). Approximately 100 g samples of *longissimus dorsi* muscle were used to determine IMF content using petroleum ether extraction 24 hours after slaughter [92]. One gram of muscle was collected to determine the pH_45_ value. The pH_45_ of the left ham was measured in the center of the *longissimus dorsi* muscle using a portable needle-tipped combination electrode (NWK binar pH-K21, CE, Germany). Color was recorded on three 10 mm diameter spots from each *longissimus dorsi* muscle eye rib surface within 2 hours following loin slicing. Indicators of lightness (L*), redness (a*), and yellowness (b*) were recorded in triplicates by a Minolta chromameter (CR-300, Minolta Camera Co., Japan) on a freshly cut surface 45 minutes postmortem, and the average value of the three spots was used.

### Extraction of RNA Samples from Muscles for Microarray Hybridization

Approximately 100 mg of frozen muscle tissues were homogenized in liquid nitrogen using a mortar and pestle under RNase-free conditions. Total RNA was extracted from the samples with Trizol-Phenol reagent (Invitrogen) according to the manufacturer’s protocols. Residue DNA was removed with DNaseI (37°C, 30 mins) followed by purification of total RNA with RNeasy Mini kit (Qiagen). RNA was quantified using NanoDrop ND-1000 spectrophotometer (NanoDrop Technologies) at 260 and 280 nm and the integrity of RNA was determined by denaturing agarose gel electrophoresis. The quality of total RNA was further assessed using an Agilent Bioanalyzer 2100 (Agilent Technologies) based on the RNA integrity number (RIN) value. All samples used for microarray analyses had an RIN value above 8.

### Microarray Hybridization

Total RNA from a total of 54 pigs at d30, d90 and d150 of age stage (nine pigs for each breed at each stage) was extracted. RNA samples from three pigs of the same breed at the same age stage were pooled as one sample for one gene-chip hybridization. Microarray data from three samples for each breed at each stage were obtained for data analysis. A total of 18 microarrays were used in the experiment, corresponding to the 18 pooled RNA samples from *longissimus dorsi* muscles. The GeneChip Porcine Genome Array (Affymetrix, Santa Clara, CA) contains 23937 probes sets interrogating 23256 transcripts, representing 20201 genes. RNA labeling and Affymetrix Gene Chip microarray hybridization were conducted according to the Affymetrix Expression Analysis Technical Manual. Array scanning and data extraction were carried out following procedures recommended by Affymetrix.

### Microarray Data Analysis

To quantify the intensities from the same probe sets on different arrays, these were scaled so that the median intensities for all arrays were the same. We then calculated the average intensity for each probe in all replicate arrays and this mean intensity was used for downstream analysis. When comparing gene expression between different breeds at the same time-point and in the same tissue, Lowess intensity dependent normalization was performed for each array pair. Z-scores were then calculated as described previously [93] and Z-scores ≥2 or ≤2 was used as the cut-off value for selection of up- or down-regulated genes. Hierarchical and K-means clustering of differentially expressed genes was done using Cluster 2.10 and viewed in TreeView 1.50 from Eisen Lab (http://rana.lbl.gov/EisenSoftware.htm).

### qPCR

Primer sequences, melting temperatures and expected product sizes for the genes analyzed are shown in Additional file 15 (Table S15). The sizes of the PCR products were confirmed using agarose gel electrophoresis (1.8%). The specificity of the PCR products was judged based on a single peak observed in dissociation/melting curves. All RNA samples prepared for gene-chip hybridization were also used in qPCR. qPCR was performed using SYBR green I nucleic acid dye on an BIO-RAD CFX96 Real-Time PCR System (BIO-RAD, Foster City, CA, USA) to quantify the target genes expression levels. Data are expressed as the ratio between expression of the target gene and that of the housekeeping gene 18s rRNA. All qPCR reactions followed this thermal profile: after an initial denaturation at 94°C for 2 minutes, amplification was performed with 40 cycles of 94°C for 30s and annealing for 40 s at temperatures specific for each target genes. For each sample, reactions were set up in triplicate to ensure the reproducibility of the results. At the end of the PCR run, melting curves were generated and analyzed to confirm non-specific amplification, and the mean value of each triplicate was used for further calculations. To calculate the mRNA expression of selective genes, the ΔCt values was used for detection of their mRNA related to internal control 18s rRNA expression using the 2^−△△Ct^ method [94].

### Cloning of the *pFLJ* Gene

To obtain the full-length cDNA sequence of *pFLJ*, RACE technology was carried out to clone the 5′-ends of *pFLJ* by using the SMARTTM RACE cDNA Amplification Kit and GeneRacer Kit (Invitrogen Biotechnology Co. Ltd., Shanghai, China). Briefly, for 5′-RACE, 5′ phosphates and the 5′ cap structure were removed from the total RNA from porcine tissues, the GeneRacer RNA Oligo sequence (5′-CGACUGGAGCACGAGGACACUGACAUGGACUGAAGGAGUAGAAA-3′) to the 5′ end of the prepared mRNA was ligated and the 5′ RACE cDNA template was then obtained by reverse-transcribing the ligated mRNA according to the manufacturer’s instructions. Four steps were required to obtain the full length of pFLJ36031 cDNA. The first reaction of PCR was performed using a combination of sm-FLG-R1 (5′-GCCACCAATGACCAAAGGCACTTGGATAA-3′) and 10*UPM using the 5′ RACE cDNA template. The PCR condition was as follows: 94°C for 2 min, 5 cycles of 94°C for 30 s and 72°C for 1.5 min, 5 cycles of 94°C for 30 s and 70°C for 1.5 min, 25 cycles of 94°C for 30 s, 65°C for 30 s and 68°C 3.0 min. Then the product was further identified using another primer (sm-FLG-R2∶5′-GCCTGATCAACGATTCCTGTGGTCTTCA-3′) that is located on the downstream of sm-FLG-R1. The PCR condition used was: 94°C 2 min, 30 cycles: 94°C 30 s 66°C 30 s and 68°C 1.5 min. The gene-specific primer sm-FLG-R1 was designed based on the *pFLJ* EST available in GenBank. The resulting PCR product obtained from this step was isolated, cloned, and sequenced. The three subsequent 5′-RACE products were gel-purified, cloned, and sequenced. By ligation of the four overlapping cDNA fragments, full-length pFLJ cDNA was obtained. Primer pairs used for qPCR were: sense: 5′-cca cct ttc cca cca ttc g-3′; antisense: 5′-agc ctc acc acg ggt tcc ag-3′.

### siRNAs Targeting *pFLJ*

Three potential siRNA target sites in *pFLJ* (FS1∶5′-aactgtcgctggccgacagca-3′; FS2∶5′-aagctgttcatgccccgcagc-3′. FS3∶5′-aaggacgtctacggctactcc-3′) were determined using the Qiagen siRNA design programme, and the sequence was BLAST-confirmed for specificity. Oligonucleotides to produce plasmid-based siRNA were cloned into pSilencer TM 4.1-CMV neo plasmid (Ambion) and all constructs were confirmed by sequencing. For RNA interference experiments, porcine intramuscular adipocytes were transfected with empty plasmid (wt), negative control siRNA (ns), or p*FLJ*-siRNA (fs1, fs2 and fs3). Transfections were performed using Lipofectamine™ 2000 (Invitrogen Life Technologies) according to the manufacturer’s protocol. A final concentration of 2000 ng/ml siRNA was used to treat the cultured intramuscular adipocytes. Negative control siRNA (Neg-siRNA, ns, 5′-acatgtgcgcagccacagctg-3′) was supplied by Ambion.

### *In vitro* Culture of Intramuscular Adipocyte Precursor Cells and Induction of Adipocytes

For *in vitro* culture of intramuscular adipocyte precursor cells, D (Duroc) ×L (Landrace)×Y (Yorkshire) pigs from d5 to d7 of age were overdosed with sodium thiopental and exsanguinated. The *longissimus dorsi* muscle was removed and porcine pre-adipocytes were prepared by previously published methods [95], [96]. Briefly, *longissimus dorsi* muscle tissue was cut with scissors into approximately1 mm sections under sterile condition and digested with collagenase type II for 45 hours, at 37°C in a 120r/min shaking water bath. The digested material collected was first centrifuged at100 g for1 min, and the resulting floating adipocytes were collected in Dulbecco’s Modified Eagle Medium (DMEM) at 37°C. The number of intramuscular pre-adipocytes isolated in suspension was determined as described previously. The preadipocytes were seeded on six-well (35-mm) tissue culture plates in complete media (DMEM/F12+10% fetal bovine serum (FBS)+100 Upenicillin+100 Ustreptomycin) and cultured at 37°C under a humidified atmosphere of 95% air and 5% carbon dioxide according to previous study [42].

Intramuscular preadipocytes were induced to differentiate into intramuscular adipocytes when the cells were completely fused and were then treated with a final concentration of 0.5 mmol/L 3-isobutyl-1-methylxanthine (IBMX), 1µmol/L dexamethasone (DEX) and 1.7µmol/L insulin of complete medium. The culture medium was changed to complete medium containing a final concentration of 10 mg/L insulin after 48 hours.

### Statistical Analysis

All experimental data of comparisons between two pig breeds were analyzed using one-way analysis of variance (ANOVA, Statistical Product and Service Solutions (SPSS) 16.0). Data are represented as means±standard error; *P<0.05 and **P<0.01 displayed here indicate statistically significant difference.

## Supporting Information

Table S1 **177 genes upregulated in longissium dorsi muscles of jinhua pig at d90 compared with that at d30 age stage (Jinhua-d90-LD-up vs d30).**
(XLS)

Table S2 **242 genes downregulated in longissium dorsi muscles of jinhua pig at d90 compared with that at d30 age stage (Jinhua-d90-LD-down vs d30).**
(XLS)

Table S3 **101 genes upregulated in longissium dorsi muscles of jinhua pig at d150 compared with that at d30 age stage (Jinhua-d150-LD-up vs d30).**
(XLS)

Table S4 **389 genes downregulated in longissium dorsi muscles of jinhua pig at d150 compared with that at d30 age stage (Jinhua-d150-LD-down vs d30).**
(XLS)

Table S5 **106 genes upregulated in longissium dorsi muscles of Landrace at d90 compared with that at d30 age stage (Landrace-d90-LD-up vs d30).**
(XLS)

Table S6 **231 genes downregulated in longissium dorsi muscles of Landrace at d90 compared with that at d30 age stage (Landrace-d90-LD-down vs d30).**
(XLS)

Table S7 **93 genes upregulated in longissium dorsi muscles of Landrace at d150 compared with that at d30 age stage (Landrace-d150-LD-up vs d30).**
(XLS)

Table S8 **383 genes downregulated in longissium dorsi muscles of Landrace at d150 compared with that at d30 age stage (Landrace-d150-LD-down vs d30).**
(XLS)

Table S9 **176 genes upregulated in longissium dorsi muscles of Jinhua pig versus Landrace at d30 of age stage (Jinhua-d30-LD-up).**
(XLS)

Table S10 **276 genes upregulated in longissium dorsi muscles of Jinhua pig versus Landrace at d90 of age stage (Jinhua-d90-LD-up).**
(XLS)

Table S11 **525 genes upregulated in longissium dorsi muscles of Jinhua pig versus Landrace at d150 of age stage (Jinhua-d150-LD-up).**
(XLS)

Table S12 **199 genes downregulated in longissium dorsi muscles of Jinhua pig versus Landrace at d30 of age stage (Jinhua-d30-LD-down).**
(XLS)

Table S13 **155 genes downregulated in longissium dorsi muscles of Jinhua pig versus Landrace at d90 of age stage (Jinhua-d90-LD-down).**
(XLS)

Table S14 **670 genes downregulated in longissium dorsi muscles of Jinhua pig versus Landrace at d150 of age stage (Jinhua-d150-LD-down).**
(XLS)

Table S15 **Primer sequences.**
(XLS)

## References

[1] Miao ZG, Wang LJ, Xu ZR, Huang JF, Wang YR (2009) Developmental changes of carcass composition, meat quality and organs in the Jinhua pig and Landrace. Animal 3: 468–473. 22444318 10.1017/S1751731108003613

[2] Guo J, Shan T, Wu T, Zhu LN, Ren Y, et al (2011) Comparisons of different muscle metabolic enzymes and muscle fiber types in Jinhua and Landrace pigs. Journal of Animal Science 89: 185–191. 20889683 10.2527/jas.2010-2983

[3] Dai FW, Feng DY, Cao QY, Ye H, Zhang CM, et al (2009) Developmental differences in carcass, meat quality and muscle fibre characteristics between the Landrace and a Chinese native pig. S Afr J Anim Sci 39: 267–273.

[4] Cameron ND, Warriss PD, Porter SJ, Enser MB (1990) Comparison of Duroc and British Landrace Pigs for Meat and Eating Quality. Meat Sci 27: 227–247. 22055287 10.1016/0309-1740(90)90053-9

[5] Wood JD, Kempster AJ, David PJ, Bovey M (1987) Observations on Carcass and Meat Quality in Duroc, Landrace and Duroc X Landrace Pigs. Anim Prod 44: 488–488.

[6] Matsakas A, Patel K (2009) Skeletal muscle fibre plasticity in response to selected environmental and physiological stimuli. Histol Histopathol 24: 611–629. 19283669 10.14670/HH-24.611

[7] Duggan DJ, Bittner M, Chen YD, Meltzer P, Trent JM (1999) Expression profiling using cDNA microarrays. Nat Genet 21: 10–14. 9915494 10.1038/4434

[8] Guo W, Wang SH, Cao HJ, Xu K, Zhang J, et al (2008) Gene microarray analysis for porcine adipose tissue: Comparison of gene expression between Chinese Xiang pig and large white. Asian Austral J Anim 21: 11–18.

[9] Pannier L, Mullen AM, Hamill RM, Stapleton PC, Sweeney T (2010) Association analysis of single nucleotide polymorphisms in DGAT1, TG and FABP4 genes and intramuscular fat in crossbred Bos taurus cattle. Meat Sci 85: 515–518. 20416823 10.1016/j.meatsci.2010.02.025

[10] Raj S, Skiba G, Weremko D, Fandrejewski H, Migdal W, et al (2010) The relationship between the chemical composition of the carcass and the fatty acid composition of intramuscular fat and backfat of several pig breeds slaughtered at different weights. Meat Sci 86: 324–330. 20665991 10.1016/j.meatsci.2010.04.037

[11] Jiang YH, Shang HW, Xu H, Ding XF, Zhao LY, et al (2010) Detection and genotyping of porcine circovirus in naturally infected pigs by oligo-microarray. Res Vet Sci 89: 133–139. 20137797 10.1016/j.rvsc.2010.01.009

[12] Paturi G, Phillips M, Kailasapathy K (2010) Comparison of functional assay and microarray analysis for determination of Lactobacillus acidophilus LAFTI L10 induced gut immune responses in mice. Food Res Int 43: 856–861.

[13] Wei L, Lijuan H, Dan L (2010) Microarray analysis of differently expressed microRNA profiles induced by UVB irradiated in mice skin. J Invest Dermatol 130: S132–S132.

[14] Serre C, Plaza C, Lebleu A, Plantivaux A, Meyrignac C, et al (2010) Microarray profiling of gene expression response to modulation of the stem cell factor/c-kit receptor signalisation pathway in human skin keratinocytes. J Invest Dermatol 130: S76–S76.

[15] Grove KL, Fried SK, Greenberg AS, Xiao XQ, Clegg DJ (2010) A microarray analysis of sexual dimorphism of adipose tissues in high-fat-diet-induced obese mice. Int J Obesity 34: 989–1000. 10.1038/ijo.2010.12 PMC3667412 20157318

[16] Campbell WG, Gordon SE, Carlson CJ, Pattison JS, Hamilton MT, et al (2001) Differential global gene expression in red and white skeletal muscle. Am J Physiol-Cell Ph 280: C763–C768. 10.1152/ajpcell.2001.280.4.C763 11245591

[17] Bai QF, McGillivray C, da Costa N, Dornan S, Evans G, et al (2003) Development of a porcine skeletal muscle cDNA microarray: analysis of differential transcript expression in phenotypically distinct muscles. Bmc Genomics 4: 8. 12611633 10.1186/1471-2164-4-8 PMC152649

[18] Zhao SH, Recknor J, Lunney JK, Nettleton D, Kuhar D, et al (2005) Validation of a first-generation long-oligonucleotide microarray for transcriptional profiling in the pig. Genomics 86: 618–625. 16216716 10.1016/j.ygeno.2005.08.001

[19] Miyagawa S, Takeishi S, Yamamoto A, Ikeda K, Matsunari H, et al (2010) Survey of glycoantigens in cells from alpha 1–3galactosyltransferase knockout pig using a lectin microarray. Xenotransplantation 17: 61–70. 20149189 10.1111/j.1399-3089.2009.00565.x

[20] Zhou GX, Wang SB, Wang ZG, Zhu XT, Shu G, et al (2010) Global comparison of gene expression profiles between intramuscular and subcutaneous adipocytes of neonatal landrace pig using microarray. Meat Sci 86: 440–450. 20573458 10.1016/j.meatsci.2010.05.031

[21] Tsai S, Cassady JP, Freking BA, Nonneman DJ, Rohrer GA, et al (2006) Annotation of the Affymetrix(1) porcine genome microarray. Anim Genet 37: 423–424. 16879364 10.1111/j.1365-2052.2006.01460.x

[22] Naraballobh W, Chomdej S, Murani E, Wimmers K, Ponsuksili S (2010) Annotation and in silico localization of the Affymetrix GeneChip Porcine Genome Array. Arch Tierzucht 53: 230–238.

[23] Jiang YZ, Zhu L, Li XW, Si T (2011) Evaluation of the Chinese indigenous pig breed Dahe and crossbred Dawu for growth and carcass characteristics, organ weight, meat quality and intramuscular fatty acid and amino acid composition. Animal 5: 1485–1492. 22440295 10.1017/S1751731111000425

[24] GarciaMacias JA, Gispert M, Oliver MA, Diestre A, Alonso P, et al (1996) The effects of cross, slaughter weight and halothane genotype on leanness and meat and fat quality in pig carcasses. Anim Sci 63: 487–496.

[25] Edwards LN, Grandin T, Engle TE, Ritter MJ, Sosnicki AA, et al (2010) The effects of pre-slaughter pig management from the farm to the processing plant on pork quality. Meat Sci 86: 938–944. 20728282 10.1016/j.meatsci.2010.07.020

[26] Latorre MA, Medel P, Fuentetaja A, Lazaro R, Mateos GG (2003) Effect of gender, terminal sire line and age at slaughter on performance, carcass characteristics and meat quality of heavy pigs. Anim Sci 77: 33–45.

[27] Latorre MA, Lazaro R, Gracia MI, Nieto M, Mateos GG (2003) Effect of sex and terminal sire genotype on performance, carcass characteristics, and meat quality of pigs slaughtered at 117 kg body weight. Meat Sci 65: 1369–1377. 22063781 10.1016/S0309-1740(03)00059-7

[28] Guo J, Shan T, Wu T, Zhu LN, Ren Y, et al (2011) Comparisons of different muscle metabolic enzymes and muscle fiber types in Jinhua and Landrace pigs. J Anim Sci 89: 185–191. 20889683 10.2527/jas.2010-2983

[29] Lefaucheur L, Milan D, Ecolan P, Le Callennec C (2004) Myosin heavy chain composition of different skeletal muscles in Large White and Meishan pigs. J Anim Sci 82: 1931–1941. 15309939 10.2527/2004.8271931x

[30] Cesar ASM, Silveira ACP, Freitas PFA, Guimaraes EC, Batista DFA, et al (2010) Influence of Chinese breeds on pork quality of commercial pig lines. Genet Mol Res 9: 727–733. 20449804 10.4238/vol9-2gmr733

[31] Eisen MB, Spellman PT, Brown PO, Botstein D (1998) Cluster analysis and display of genome-wide expression patterns. Proc Natl Acad Sci U S A 95: 14863–14868. 9843981 10.1073/pnas.95.25.14863 PMC24541

[32] Chen HB, Li CC, Fang MD, Zhu MJ, Li XY, et al (2009) Understanding Haemophilus parasuis infection in porcine spleen through a transcriptomics approach. Bmc Genomics 10: 64. 19196461 10.1186/1471-2164-10-64 PMC2660370

[33] Shan TZ, Wang YZ, Liu YJ, Liu JX, Feng J, et al (2006) Developmental expression of the lipoprotein lipase gene in porcine subcutaneous adipose tissue. J Anim Feed Sci 15: 621–629.

[34] Pulawa LK, Jensen DR, Jung DY, Hong EG, Coates AM, et al (2007) Muscle-specific lipoprotein lipase deletion increases insulin action in skeletal muscle with resultant excess adipose tissue deposition and systemic insulin resistance. Diabetes 56: A340–A340.

[35] Costabile G, Annuzzi G, Di Marino L, De Natale C, Giacco R, et al (2011) Fasting and post-prandial adipose tissue lipoprotein lipase and hormone-sensitive lipase in obesity and Type 2 diabetes. J Endocrinol Invest 34: E110–E114. 20926921 10.1007/BF03347469

[36] Shan T, Wu T, Reng Y, Wang Y (2009) Breed difference and regulation of the porcine adipose triglyceride lipase and hormone sensitive lipase by TNF alpha. Anim Genet 40: 863–870. 19496768 10.1111/j.1365-2052.2009.01927.x

[37] Shen WJ, Yu ZX, Patel S, Jue D, Kraemer FB (2008) Hormone-sensitive lipase (HSL) modulates adipose metabolism through PPAR-Gamma. Diabetes 57: A390–A390. 10.1016/j.bbalip.2010.10.001 PMC2998198 20950707

[38] Schweiger M, Schreiber R, Haemmerle G, Lass A, Fledelius C, et al (2006) Adipose triglyceride lipase and hormone-sensitive lipase are the major enzymes in adipose tissue triacylglycerol catabolism. J Biol Chem 281: 40236–40241. 17074755 10.1074/jbc.M608048200

[39] Zhao SM, Wang J, Song XL, Zhang X, Ge CR, et al (2010) Impact of dietary protein on lipid metabolism-related gene expression in porcine adipose tissue. Nutrition & Metabolism 7: 6. 20205889 10.1186/1743-7075-7-6 PMC2827416

[40] Reiter SS, Halsey CHC, Stronach BM, Bartosh JL, Owsley WF, et al (2007) Lipid metabolism related gene-expression profiling in liver, skeletal muscle and adipose tissue in crossbred Duroc and Pietrain Pigs. Comp Biochem Phys D 2: 200–206. 10.1016/j.cbd.2007.04.008 20483293

[41] Ding ST, Schinckel AP, Weber TE, Mersmann HJ (2000) Expression of porcine transcription factors and genes related to fatty acid metabolism in different tissues and genetic populations. J Anim Sci 78: 2127–2134. 10947099 10.2527/2000.7882127x

[42] Shan TZ, Ren Y, Wu T, Liu CX, Wang YZ (2009) Regulatory Role of Sirt1 on the Gene Expression of Fatty Acid-Binding Protein 3 in Cultured Porcine Adipocytes. J Cell Biochem 107: 984–991. 19479941 10.1002/jcb.22203

[43] Mostyn A, Williams PJ, Litten JC, Perkins KS, Corson AM, et al (2007) Differences in fatty acid-binding protein (FABP) 3 and 4 mRNA expression in skeletal muscle and subcutaneous adipose tissue between normal-birth-weight and low- and high-birth-weight porcine offspring at days 7 and 14 of postnatal life. P Nutr Soc 66: 57a–57a.

[44] Chmurzynska A (2006) The multigene family of fatty acid-binding proteins (FABPs): Function, structure and polymorphism. J Appl Genetics 47: 39–48. 16424607 10.1007/BF03194597

[45] Ma J, Mollsten A, Falhammar H, Brismar K, Dahlquist G, et al (2007) Genetic association analysis of the adiponectin polymorphisms in type 1 diabetes with and without diabetic nephropathy. J Diabetes Complicat 21: 28–33. 17189871 10.1016/j.jdiacomp.2006.03.002

[46] Gomez-Ruiz A, Milagro FI, Campion J, Martinez JA, Miguel C (2010) Caveolin Expression and Activation in Retroperitoneal and Subcutaneous Adipocytes: Influence of a High-Fat Diet. J Cell Physiol 225: 206–213. 20506294 10.1002/jcp.22241

[47] Lopez IP, Milagro FI, Marti A, Moreno-Aliaga MJ, Martinez JA, et al (2005) High-fat feeding period affects gene expression in rat white adipose tissue. Mol Cell Biochem 275: 109–115. 16335790 10.1007/s11010-005-1082-z

[48] Dagher G, Donne N, Klein C, Ferre P, Dugail I (2003) HDL-mediated cholesterol uptake and targeting to lipid droplets in adipocytes. J Lipid Res 44: 1811–1820. 12867544 10.1194/jlr.M300267-JLR200

[49] Wang D, Wang N, Li N, Li H (2009) Identification of differentially expressed proteins in adipose tissue of divergently selected broilers. Poultry Sci 88: 2285–2292. 19834077 10.3382/ps.2009-00190

[50] Schmitt B, Fluck M, Decombaz J, Kreis R, Boesch C, et al (2003) Transcriptional adaptations of lipid metabolism in tibialis anterior muscle of endurance-trained athletes. Physiol Genomics 15: 148–157. 14565968 10.1152/physiolgenomics.00089.2003

[51] Cotter DG, d'Avignon DA, Wentz AE, Weber ML, Crawford PA (2011) Obligate Role for Ketone Body Oxidation in Neonatal Metabolic Homeostasis. J Biol Chem 286: 6902–6910. 21209089 10.1074/jbc.M110.192369 PMC3044945

[52] Bayer S, Birkemeyer C, Ballschmiter M (2011) A nitrilase from a metagenomic library acts regioselectively on aliphatic dinitriles. Appl Microbiol Biot 89: 91–98. 10.1007/s00253-010-2831-9 20725724

[53] Yang HJ, Zhou ZH, Zhang HR, Chen M, Li JY, et al (2010) Shotgun proteomic analysis of the fat body during metamorphosis of domesticated silkworm (Bombyx mori). Amino Acids 38: 1333–1342. 19730979 10.1007/s00726-009-0342-8

[54] Soni KG, Lehner R, Metalnikov P, O'Donnell P, Semache M, et al (2004) Carboxylesterase 3 (EC 3.1.1.1) is a major adipocyte lipase. J Biol Chem 279: 40683–40689. 15220344 10.1074/jbc.M400541200

[55] Kaphalia BS, Ansari GAS (2001) Purification and characterization of rat hepatic microsomal low molecular weight fatty acid ethyl ester synthase and its relationship to carboxylesterases. J Biochem Mol Toxic 15: 165–171. 10.1002/jbt.14 11424227

[56] Kaphalia BS, Fritz RR, Ansari GAS (1997) Purification and characterization of rat liver microsomal fatty acid ethyl and 2-chloroethyl ester synthase and their relationship with carboxylesterase (pI 6.1). Chem Res Toxicol 10: 211–218. 9049433 10.1021/tx960079e

[57] Van den Maagdenberg K, Claeys E, Stinckens A, Buys N, De Smet S (2007) Effect of age, muscle type, and insulin-like growth factor-II genotype on muscle proteolytic and lipolytic enzyme activities in boars. J Anim Sci 85: 952–960. 17202393 10.2527/jas.2006-563

[58] Symonds ME, Pearce S, Bispham J, Gardner DS, Stephenson T (2004) Timing of nutrient restriction and programming of fetal adipose tissue development. P Nutr Soc 63: 397–403. 10.1079/pns2004366 15373949

[59] Fenton JI, Nunez NP, Yakar S, Perkins SN, Hord NG, et al (2009) Diet-induced adiposity alters the serum profile of inflammation in C57BL/6N mice as measured by antibody array. Diabetes Obes Metab 11: 343–354. 19267713 10.1111/j.1463-1326.2008.00974.x PMC5488284

[60] Kallio P, Tolppanen AM, Kolehmainen M, Poutanen K, Lindstrom J, et al (2009) Association of sequence variations in the gene encoding insulin-like growth factor binding protein 5 with adiponectin. Int J Obesity 33: 80–88. 10.1038/ijo.2008.196 18957933

[61] Wang HB, Li H, Wang QG, Zhang XY, Wang SZ (2007) Profiling of chicken adipose tissue gene expression by genome array. Bmc Genomics 2007 8: 193. 10.1186/1471-2164-8-193 PMC1914355 17594506

[62] Donkor J, Sparks LM, Xie H, Smith SR, Reue K (2008) Adipose tissue lipin-1 expression is correlated with peroxisome proliferator-activated receptor alpha gene expression and insulin sensitivity in healthy young men. J Clin Endocr Metab 93: 233–239. 17925338 10.1210/jc.2007-1535 PMC2190746

[63] Ishimoto K (2011) Lipin 1 in Lipid Metabolism. Yakugaku Zasshi 131: 1189–1194. 21804322 10.1248/yakushi.131.1189

[64] Evock-Clover CM, Poch SM, Richards MP, Ashwell CM, McMurtry JP (2002) Expression of an uncoupling protein gene homolog in chickens. Comparative Biochemistry and Physiology a-Molecular & Integrative Physiology 133: 345–358. 10.1016/s1095-6433(02)00113-7 12208305

[65] Femia AP, Luceri C, Toti S, Giannini A, Dolara P, et al (2010) Gene expression profile and genomic alterations in colonic tumours induced by 1,2-dimethylhydrazine (DMH) in rats. Bmc Cancer 10: 194. 20459814 10.1186/1471-2407-10-194 PMC2877689

[66] Barroso E, Rodriguez-Calvo R, Serrano-Marco L, Astudillo AM, Balsinde J, et al (2011) The PPAR beta/delta Activator GW501516 Prevents the Down-Regulation of AMPK Caused by a High-Fat Diet in Liver and Amplifies the PGC-1 alpha-Lipin 1-PPAR alpha Pathway Leading to Increased Fatty Acid Oxidation. Endocrinology 152: 1848–1859. 21363937 10.1210/en.2010-1468

[67] Yuan YA, Shi XE, Liu YG, Yang GS (2011) FoxO1 regulates muscle fiber-type specification and inhibits calcineurin signaling during C2C12 myoblast differentiation. Mol Cell Biochem 348: 77–87. 21080037 10.1007/s11010-010-0640-1

[68] Wu AL, Kim JH, Zhang CB, Unterman TG, Chen J (2008) Forkhead box protein O1 negatively regulates skeletal myocyte differentiation through degradation of mammalian target of rapamycin pathway components. Endocrinology 149: 1407–1414. 18079193 10.1210/en.2007-1470 PMC2275355

[69] Cassano M, Dellavalle A, Tedesco FS, Quattrocelli M, Crippa S, et al (2011) Alpha sarcoglycan is required for FGF-dependent myogenic progenitor cell proliferation in vitro and in vivo. Development 138: 4523–4533. 21903674 10.1242/dev.070706

[70] Kabaeva Z, Meekhof KE, Michele DE (2011) Sarcolemma instability during mechanical activity in Large(myd) cardiac myocytes with loss of dystroglycan extracellular matrix receptor function. Hum Mol Genet 20: 3346–3355. 21628317 10.1093/hmg/ddr240 PMC3153301

[71] Gardner KL, Sanford JL, Mays TA, Rafael-Fortney JA (2006) CASK localizes to nuclei in developing skeletal muscle and motor neuron culture models and is agrin-independent. J Cell Physiol 206: 196–202. 15965905 10.1002/jcp.20449

[72] Siders JL, Hainsey TA, Murnaghan S, Wilson JB, Rafael JA (2001) Developmental and overexpression studies of CASK in skeletal muscle. Am J Hum Genet 69: 639–639.

[73] Verner J, Humpolicek P, Knoll A (2007) Impact of MYOD family genes on pork traits in Large White and Landrace pigs. J Anim Breed Genet 124: 81–85. 17488358 10.1111/j.1439-0388.2007.00639.x

[74] Pas MFWT, Verburg FJ, Gerritsen CLM, de Greef KH (2000) Messenger ribonucleic acid expression of the MyoD gene family in muscle tissue at slaughter in relation to selection for porcine growth rate. J Anim Sci 78: 69–77. 10682804 10.2527/2000.78169x

[75] Wang Q, Young TM, Mathews MB, Pe'ery T (2007) Developmental regulators containing the I-mfa domain interact with T cyclins and Tat and modulate transcription. J Mol Biol 367: 630–646. 17289077 10.1016/j.jmb.2007.01.020 PMC1868487

[76] Li ZC, Zhao BP, Kim YS, Hu CY, Yang JZ (2010) Administration of a Mutated Myostatin Propeptide to Neonatal Mice Significantly Enhances Skeletal Muscle Growth. Mol Reprod Dev 77: 76–82. 19743472 10.1002/mrd.21111

[77] Patruno M, Caliaro F, Maccatrozzo L, Sacchetto R, Martinello T, et al (2008) Myostatin shows a specific expression pattern in pig skeletal and extraocular muscles during pre- and post-natal growth. Differentiation 76: 168–181. 17573916 10.1111/j.1432-0436.2007.00189.x

[78] Newcom DW, Stalder KJ, Baas TJ, Goodwin RN, Parrish FC, et al (2004) Breed differences and genetic parameters of myoglobin concentration in porcine longissimus muscle. J Anim Sci 82: 2264–2268. 15318723 10.2527/2004.8282264x

[79] Rosell CM, Flores M, Toldra F (1996) Myoglobin as an endogenous inhibitor of proteolytic muscle enzymes. J Agr Food Chem 44: 3453–3456.

[80] Hayes AJ, Benjamin M, Ralphs JR (2001) Extracellular matrix in development of the intervertebral disc. Matrix Biol 20: 107–121. 11334712 10.1016/s0945-053x(01)00125-1

[81] Geyer CB, Inselman AL, Sunman JA, Bornstein S, Handel MA, et al (2009) A missense mutation in the Capza3 gene and disruption of F-actin organization in spermatids of repro32 infertile male mice. Dev Biol 330: 142–152. 19341723 10.1016/j.ydbio.2009.03.020 PMC2688473

[82] Soeno Y, Hayakawa K, Obinata T (1998) Effects of exogenous beta-actinin (CapZ) on actin filamentous structures in cultured muscle cells. Zool Sci 15: 217–222.

[83] Gray S, Feinberg MW, Hull S, Kuo CT, Watanabe M, et al (2002) The Kruppel-like factor KLF15 regulates the insulin-sensitive glucose transporter GLUT4. J Biol Chem 277: 34322–34328. 12097321 10.1074/jbc.M201304200

[84] Bernot D, Barruet E, Poggi M, Bonardo B, Alessi MC, et al (2010) Down-regulation of Tissue Inhibitor of Metalloproteinase-3 (TIMP-3) Expression Is Necessary for Adipocyte Differentiation. J Biol Chem 285: 6508–6514. 20056610 10.1074/jbc.M109.078444 PMC2825446

[85] Inuzuka H, Wakao H, Masuho Y, Muramatsu M, Tojo H, et al (1999) cDNA cloning and expression analysis of mouse zf9, a Kruppel-like transcription factor gene that is induced by adipogenic hormonal stimulation in 3T3-L1 cells. Bba-Gene Struct Expr 1447: 199–207. 10.1016/s0167-4781(99)00161-x 10542316

[86] Grizard J, Picard B, Dardevet D, Balage M, Rochon C (1999) Regulation of muscle growth and development. Protein Metabolism and Nutrition 96: 177–201.

[87] Kemp TJ, Sadusky TJ, Saltisi F, Carey N, Moss J, et al (2000) Identification of Ankrd2, a novel skeletal muscle gene coding for a stretch-responsive ankyrin-repeat protein. Genomics 66: 229–241. 10873377 10.1006/geno.2000.6213

[88] Jiang WQ, Chang ACM, Satoh M, Furuichi Y, Tam PPL, et al (2000) The distribution of stanniocalcin 1 protein in fetal mouse tissues suggests a role in bone and muscle development. J Endocrinol 165: 457–466. 10810309 10.1677/joe.0.1650457

[89] Serlachius M, Andersson LC (2004) Upregulated expression of stanniocalcin-1 during adipogenesis. Exp Cell Res 296: 256–264. 15149855 10.1016/j.yexcr.2004.02.016

[90] Ao WY, Pilgrim D (2000) Caenorhabditis elegans UNC-45 is a component of muscle thick filaments and colocalizes with myosin heavy chain B, but not myosin heavy chain A. J Cell Biol. 148: 375–384. 10.1083/jcb.148.2.375 PMC2174295 10648570

[91] Soranzo N, Rendon A, Gieger C, Jones CI, Watkins NA, et al (2009) A novel variant on chromosome 7q22.3 associated with mean platelet volume, counts, and function. Blood 113: 3831–3837. 19221038 10.1182/blood-2008-10-184234 PMC2714088

[92] Fortin A, Robertson WM, Tong AKW (2005) The eating quality of Canadian pork and its relationship with intramuscular fat. Meat Sci 69: 297–305. 22062822 10.1016/j.meatsci.2004.07.011

[93] Cheadle C, Vawter MP, Freed WJ, Becker KG (2003) Analysis of microarray data using Z score transformation. J Mol Diagn 5: 73–81. 12707371 10.1016/S1525-1578(10)60455-2 PMC1907322

[94] Livak KJ, Schmittgen TD (2001) Analysis of relative gene expression data using real-time quantitative PCR and the 2(T)(-Delta Delta C) method. Methods 25: 402–408. 11846609 10.1006/meth.2001.1262

[95] Gardan D, Gondret F, Louveau I (2006) Lipid metabolism and secretory function of porcine intramuscular adipocytes compared with subcutaneous and perirenal adipocytes. Am J Physiol Endocrinol Metab 291: E372–380. 16705057 10.1152/ajpendo.00482.2005

[96] Gardan D, Louveau L, Gondret F (2007) Adipocyte- and heart-type fatty acid binding proteins are both expressed in subcutaneous and intramuscular porcine (Sus scrofa) adipocytes. Comp Biochem Phys B 148: 14–19. 10.1016/j.cbpb.2007.03.017 17600747

